# Accelerating towards *P. vivax* elimination with a novel serological test-and-treat strategy: a modelling case study in Brazil

**DOI:** 10.1016/j.lana.2023.100511

**Published:** 2023-05-19

**Authors:** Narimane Nekkab, Thomas Obadia, Wuelton M. Monteiro, Marcus V.G. Lacerda, Michael White, Ivo Mueller

**Affiliations:** a Institut Pasteur, Université Paris Cité, G5 Épidémiologie et Analyse des Maladies Infectieuses, Paris, France; b Swiss Tropical and Public Health Institute, Allschwil, Switzerland; c University of Basel, Basel, Switzerland; d Institut Pasteur, Université Paris Cité, Bioinformatics and Biostatistics Hub, Paris, France; e Escola Superior de Ciências da Saúde, Universidade do Estado do Amazonas, Manaus, Brazil; f Diretoria de Ensino e Pesquisa, Fundação de Medicina Tropical Dr. Heitor Vieira Dourado, Manaus, Brazil; g Instituto Leônidas e Maria Deane, Fundação Oswaldo Cruz, Manaus, Brazil; h Population Health & Immunity Division, Walter and Eliza Hall Institute of Medical Research, Parkville, Australia; i Department of Medical Biology, University of Melbourne, Melbourne, Australia

**Keywords:** Plasmodium vivax, Malaria, Mathematical modelling, Serological diagnostics, Radical cure, Elimination, Mass screening, Treatment

## Abstract

**Background:**

*Plasmodium vivax* malaria is challenging to control and eliminate. Treatment with radical cure drugs fails to target the hidden asymptomatic and hypnozoite reservoirs in populations. *Pv*SeroTAT, a novel serological test-and-treat intervention using a serological diagnostic to screen hypnozoite carriers for radical cure eligibility and treatment, could accelerate *P. vivax* elimination.

**Methods:**

Using a previously developed mathematical model of *P. vivax* transmission adapted to the Brazilian context as a case study for implementation, we evaluate the public health impact of various deployment strategies of *Pv*SeroTAT as a mass campaign. We compare relative reductions in prevalence, cases averted, glucose-6-phosphate dehydrogenase (G6PD) tests, and treatment doses of *Pv*SeroTAT campaigns to strengthened case management alone or mass drug administration (MDA) campaigns across different settings.

**Findings:**

Deploying a single round of *Pv*SeroTAT with 80% coverage to treat cases with a high efficacy radical cure regimen with primaquine is predicted to reduce point population prevalence by 22.5% [95% UI: 20.2%–24.8%] in a peri-urban setting with high transmission and by 25.2% [95% UI: 9.6%–42.2%] in an occupational setting with moderate transmission. In the latter example, while a single *Pv*SeroTAT achieves 9.2% less impact on prevalence and averts 300 less cases per 100,000 than a single MDA (25.2% [95% UI: 9.6%–42.2%] point prevalence reduction versus 34.4% [95% UI: 24.9%–44%]), *P*vSeroTAT requires 4.6 times less radical cure treatments and G6PD tests. Layering strengthened case management and deploying four rounds of *Pv*SeroTAT six months apart is predicted to reduce point prevalence by a mean of 74.1% [95% UI: 61.3%–86.3%] or more in low transmission settings with less than 10 cases per 1000 population.

**Interpretation:**

Modelling predicts that mass campaigns with *Pv*SeroTAT are predicted to reduce *P. vivax* parasite prevalence across a range of transmission settings and require fewer resources than MDA. In combination with strengthened case management, mass campaigns of serological test-and-treat interventions can accelerate towards *P. vivax* elimination.

**Funding:**

This project was funded in part by the 10.13039/100000865Bill and Melinda Gates Foundation and the 10.13039/501100000925National Health and Medical Research Council.

**Research in context**

**Evidence before this study**

Mathematical modelling informed by clinical and epidemiological data have improved our understanding of *Plasmodium vivax* malaria transmission dynamics and the potential public health impact of interventions. Some *P. vivax* parasites during primary infections develop into hypnozoites that remain dormant until they activate and cause relapses weeks or months after the primary infection. Clinical trials have shown that relapses account for up to 80% of PCR-detectable blood-stage infections in children and modelling predicts that *P. vivax* elimination would be slow without directly targeting these clinically silent stages.

We searched PubMed using the terms *P. vivax* AND serolo∗ AND (intervention OR tool OR diagnostic) to identify serology-based interventions for *P. vivax* malaria and another search using the terms *P. vivax* AND (epidemio∗ OR trend∗ OR prevalence OR incidence) AND Brazil to understand current trends. A novel serological diagnostic tool that measures antibody levels to biomarkers of past infection has been validated to observational cohort data using a machine learning algorithm to detect infection within the past nine months with 80% sensitivity and 80% specificity. Modelling has shown that a serological screen-and-treat intervention is more effective than blood-stage mass screen-and-treat strategies and only marginally less effective than mass drug administration, with diagnostic sensitivity being the key driver of impact. Treatment with a blood-stage clearance drug such as chloroquine and liver-stage drugs such as primaquine can provide radical cure of all parasites with different estimated levels of clearance efficacy. *P. vivax* transmission in the Brazilian Amazon Region is heterogeneous and an increase in burden since 2015 has been observed for indigenous populations and children and importations in miners or migrants in border states. Brazil, despite implementing strong case management of *P. vivax* with radical cure, has recently launched a malaria elimination plan, for which no new technologies were addressed.

**Added value of this study**

*P. vivax* malaria is particularly challenging to eliminate and current tools and interventions fail to disrupt transmission. Novel tools are needed to accelerate elimination efforts by targeting the entire parasite reservoirs, in particular in asymptomatic carriers that contribute significantly to onward transmission. A novel mass campaign intervention, *Pv*SeroTAT, combines both serological testings to screen for potential parasite carriers and improved radical cure treatment to prevent future relapses. Using a robust mathematical model that captures *P. vivax* transmission dynamics, we demonstrate the public health benefit of implementing *Pv*SeroTAT across heterogeneous endemic transmission settings and for different deployment strategies. Additionally, our study demonstrates the added benefit of combining strengthened case management and *Pv*SeroTAT mass campaigns.

**Implications of all the available evidence**

Our findings identified optimal deployment strategies for novel serological test-and-treat interventions that can accelerate *P. vivax* elimination efforts. These modelling findings can inform both clinical trial design and future programmatic implementation in terms of site selection, timing and the number of rounds, and expected impact estimates. Our modelling results can be further validated by clinical evidence to accelerate regulatory approval of novel serological diagnostic tools for *P. vivax*. Modelling can support the adoption of such tools by policymakers and guide national implementation strategies.

## Introduction

Despite ongoing efforts to improve access to radical cure, case management of clinical *P. vivax* malaria cases will undoubtedly miss asymptomatic carriers, which can constitute up to 90% of all *P. vivax* infections.^1^ Diagnosis and treatment of *P. vivax* malaria is further complicated by a high proportion of parasites present in deep organs such as the spleen, liver and bone marrow rather than in circulation.^2^ Individuals with untreated asymptomatic infections contribute to transmission with early development of gametocytes during both primary and relapsing infection.^1^^,^^3^, ^4^, ^5^ Radical cure drugs to prevent relapses are not widely available in endemic regions, making it particularly challenging to eliminate *P. vivax*.^6^^,^^7^ New diagnostic tools to identify cases and treat individuals with radical cure drugs are needed to interrupt transmission and accelerate elimination.

Healthcare systems are required to safely deliver radical cure with 8-aminoquinolone drugs such as primaquine or tafenoquine. The World Health Organization (WHO) recommends, whenever possible, glucose-6-phosphate dehydrogenase (G6PD) testing before administration of primaquine to prevent haemolysis among G6PD deficient individuals, however, the test is not routinely used in most countries.^8^ Diagnostic testing of G6PD deficiency has been shown to be cost-effective in modelling analyses and in preventing primaquine-related hospitalisations.^9^^,^^10^ Yet the dose at which primaquine should be given is unclear. While most countries, including Brazil, recommend a total primaquine dose of 3.5 mg/kg, a recent trial demonstrated that doubling the dose to 7.0 mg/kg given over 14 days increase recurrence-free percentage at day 168 to 86% compared to 59% among those who received the lower total dose regimen.^11^ These results however reflect directly observed therapy with potentially higher adherence than real-world compliance, particularly for the 14-day regimen. While strengthening case management and improving treatment adherence have the potential to reduce transmission, without other interventions they will not lead to elimination because clinical cases represent only a proportion of *P. vivax* parasite carriers in populations.^11^^,^^12^ The population-level impact of case management with higher efficacy primaquine regimens on *P. vivax* transmission has not yet been demonstrated.

Mass campaign interventions with primaquine could be considered for targeting asymptomatic carriers and accelerating elimination efforts. Mass-drug-administration (MDA) with primaquine has been used during malaria elimination programs (e.g. in China, the Caucasus region, and Central Asia),^13^^,^^14^ however, there is no conclusive evidence supporting programmatic implementation for reducing *P. vivax* transmission.^15^ MDA is associated with significant overtreatment of the population and exposes some individuals to 8-aminoquinolone-induced haemolysis if there is no screening of G6PDd individuals.^3^^,^^14^^,^^16^ Risk of haemolysis renders MDA with 8-aminoquinolones logistically challenging and thus not acceptable for many malaria programs. Mass-screen-and-treat (MSAT) campaigns testing for blood stage parasites with a rapid diagnostic test (RDT) or light microscopy have little impact on *P. vivax* transmission due to both their relatively low sensitivity to detect low density blood stage infection and inability to detect latent liver stage infections and cryptic infections in the haematopoietic niche of bone marrow and the spleen.^17^^,^^18^

To address these challenges, a novel tool based on antibody titers of validated serological markers of exposure has been developed to identify previous *P. vivax* infection.^19^ This first-generation serological diagnostic tool using machine learning algorithms is validated to identify those with a PCR-detectable blood stage infection in the previous nine months with 80% sensitivity and 80% specificity. Such a serological diagnostic could thus be used to screen and treat individuals with recent *P. vivax* exposure and thus likely hypnozoite and/or cryptic infections in mass campaign interventions. Initial target product profile (TPP) modelling has suggested the importance of achieving very high diagnostic sensitivity to achieve similar reductions in *P. vivax* prevalence compared to MDA. However, increased sensitivity is traded-off against reduced specificity, and a higher proportion of overtreatment where false positives are given radical cure.^20^ What is currently not yet well understood is the potential population-level impact of this novel intervention in real-world settings in addition to what would be the most favourable deployment strategies to achieve greatest transmission reduction and acceleration to pre-elimination phases.

While the risk of primaquine-induced haemolysis has prevented uptake in other *P. vivax* endemic countries in South-East Asia and the Pacific,^21^ Brazil has demonstrated how strong management of clinical cases with primaquine without G6PD testing can result in reduction in transmission across the country. Brazil's intensive passive case detection and management of clinical *P. vivax* cases contributed to transmission reductions since 2000.^22^, ^23^, ^24^ Additionally, chloroquine and primaquine are provided free of charge by the government for positive diagnoses and delivered at all levels of the healthcare system in urban and rural communities.^25^ Since approving tafenoquine for radical cure in 2019, Brazil has also moved to train, test, and evaluate G6PD deficiency diagnostic tools with the aim of widespread use in the coming years.^26^^,^^27^ Yet despite significant reduction in *P. vivax* in recent years, there is still wide heterogeneity of transmission across the endemic Amazon region of Brazil with outbreaks, importations, and drug resistance threatening progress toward elimination milestones by 2030.^28^^,^^29^

Here we consider how mass campaign interventions targeting asymptomatic *P. vivax* infected individuals with a serological diagnostic screening and treatment can further accelerate endemic settings to reach pre-elimination phases. We use Brazil as an example of a setting with heterogeneous transmission and a healthcare system that can support mass test-and-treat campaigns with strengthened case management using primaquine and G6PD testing. Using our previously developed model of *P. vivax* transmission adapted to Brazilian settings, we thus consider the public health impact of *Pv*SeroTAT interventions screening the population for seropositive cases and treating them with a higher efficacy primaquine radical cure regimen after G6PD testing.^12^ Strengthened case management alone and MDA campaigns are modelled as comparators. We model the reduction in total population *P. vivax* PCR prevalence and the resources, including treatment courses and tests required to achieve these gains, for multiple rounds, timing of deployment, and layering interventions.

## Methods

### *P. vivax* transmission model

Our study uses a mathematical model to capture the complex mechanistic dynamics of *P. vivax* malaria transmission to estimate and compare the potential public health impact of novel interventions with radical cure treatment, a serological diagnostic tool for screening, and G6PD testing. Modelling facilitates hypothetical scenario testing and generates evidence to guide design and planning of novel interventions. We simulate a range of scenarios using a previously described individual-based *P. vivax* transmission model calibrated to Brazilian settings (Table 1).^5^^,^^12^^,^^20^ The detailed individual-based simulation model of *P. vivax* transmission with both human and mosquito compartments was previously calibrated to data from epidemiological studies in Papua New Guinea and the Solomon Islands.^5^ This model captures the contributions of relapse infections which are vital for *P. vivax* transmission dynamics. Several interventions are included: vector control (e.g. long-lasting insecticidal nets) and treatment pathways for radical cure with chloroquine, primaquine, and tafenoquine, accounting for differences in adherence and efficacy.^5^^,^^12^ Pathways assess treatment eligibility via age, pregnancy and lactating status, and G6PD phenotypic activity in men and women, drug metabolism, and drug efficacy against blood-stage parasite clearance. *Pv*SeroTAT interventions are implemented to screen individuals with *P. vivax* infection within the previous nine months for the various treatment pathways.^20^ Performance of the serological diagnostic tool modelled are based on sensitivity and specificity estimates from previous studies.^19^^,^^20^ Calibration for Brazilian settings of baseline incidence, G6PD deficiency prevalence, and occupational exposure risk are detailed in the Supplementary Materials and in a previous publication by the authors.^12^ The model is publicly available online at https://github.com/MWhite-InstitutPasteur/Pvivax_TQ_IBM.

**Table 1:** Simulated scenarios.

	Intervention	Rounds	Months between rounds	Coverage (%)	seven-day PQ adherence (%)	seven-day PQ efficacy (%)	G6PD testing	Sensitivity (%)	Specificity (%)
**S**_**0**_	Baseline CM			0.95	0.667	0.714	No		
**S**_**1**_	Strengthened CM			0.95	0.80	0.80	Yes		
**S**_**2**_	*Pv*SeroTAT	1		0.80	0.80	0.80	Yes	0.80	0.80
**S**_**2a**_	*Pv*SeroTAT	1		0.80	0.80	0.80	Yes	0.60	0.90
**S**_**2b**_	*Pv*SeroTAT	1		0.80	0.80	0.80	Yes	0.90	0.60
**S**_**3a**_	*Pv*SeroTAT	2	6	0.80	0.80	0.80	Yes	0.80	0.80
**S**_**3b**_	*Pv*SeroTAT	2	12	0.80	0.80	0.80	Yes	0.80	0.80
**S**_**3c**_	*Pv*SeroTAT	3	6	0.80	0.80	0.80	Yes	0.80	0.80
**S**_**3d**_	*Pv*SeroTAT	3	12	0.80	0.80	0.80	Yes	0.80	0.80
**S**_**3e**_	*Pv*SeroTAT	4	6	0.80	0.80	0.80	Yes	0.80	0.80
**S**_**3f**_	*Pv*SeroTAT	4	12	0.80	0.80	0.80	Yes	0.80	0.80
**S**_**4**_	MDA	1		0.80	0.80	0.80	Yes		
**S**_**4a**_	MDA	2	6	0.80	0.80	0.80	Yes		
**S**_**4b**_	MDA	2	12	0.80	0.80	0.80	Yes		
**S**_**4c**_	MDA	3	6	0.80	0.80	0.80	Yes		
**S**_**4d**_	MDA	3	12	0.80	0.80	0.80	Yes		
**S**_**4e**_	MDA	4	6	0.80	0.80	0.80	Yes		
**S**_**4f**_	MDA	4	12	0.80	0.80	0.80	Yes		
**S**_**5a**_	*Pv*SeroTAT	1 in mid-season		0.80	0.80	0.80	Yes	0.80	0.80
**S**_**5b**_	*Pv*SeroTAT	1 in high season		0.80	0.80	0.80	Yes	0.80	0.80
**S**_**6a**_	S_1_ + S_3a_	5 years + 2 rounds	6	0.80	0.80	0.80	Yes	0.80	0.80
**S**_**6b**_	S_1_ + S_3e_	5 years + 4 rounds	6	0.80	0.80	0.80	Yes	0.80	0.80

CM: case management.

*Pv*SeroTAT: serological test-and-treat for *P. vivax*.

MDA: mass-drug-administration.

PQ: primaquine.

G6PD: glucose-6-phosphate dehydrogenase.

### Intervention scenarios

Two case management (CM) interventions and twenty different mass campaigns including *Pv*SeroTAT and MDA are simulated. All individuals covered in the MDA campaign are administered chloroquine and only those eligible and G6PD normal are administered primaquine. For *Pv*SeroTAT campaigns, chloroquine is given to all individuals who are then screened for a previous infection within the last nine months and when seropositive, are also tested for G6PD phenotypic activity. Seropositive and G6PD normal individuals are administered primaquine. These interventions are further described in the Supplementary Materials. For all mass campaign interventions, we assume a background baseline scenario (S_0_) for case management. Interventions vary by their levels of coverage, rates of adherence to primaquine regimens, use of G6PD testing, and *Pv*SeroTAT diagnostic performance. Different deployment strategies are considered by varying the timing of initial deployment during the transmission season, number of rounds, and timing between rounds varying from six to 12 months apart.

For all scenarios, we assume all individuals older than six months are eligible for radical cure treatment except for pregnant women. The baseline scenario S_0_ assume 95% coverage of all symptomatic cases with no G6PD testing, 66.7% adherence to a radical cure regimen with seven-day 3.5 mg/kg dose primaquine and three-day chloroquine. Baseline radical cure assumes 94.8% blood stage parasite clearance co-administration efficacy and 71.4% liver stage parasite clearance efficacy for full adherence. Strengthened case management (S_1_) and mass campaigns assume improved radical cure with G6PD testing, 80% treatment adherence, 94.8% blood stage parasite clearance co-administration efficacy, and 80% liver stage parasite clearance efficacy. Only individuals with intermediate and normal G6PD activity >30% are eligible for the high efficacy dose while deficient individuals with ≤30% G6PD activity received only a chloroquine course. S_1_ assumes 95% coverage of all symptomatic cases. Mass campaigns assume 80% population coverage for each round with an individual–level correlation across consecutive rounds.

All impact indicators are calculated for each simulation and we report mean estimates with 95% uncertainty intervals using the 2.5% and 97.5% percentiles for 100 model stochastic simulations. The intervention effect size is measured by calculating the percent change between two time points (baseline and intervention follow-up) for *P. vivax* parasite prevalence in the population for all ages (zero to 80 years) detectable by polymerase chain reaction methods (*Pv*PR_PCR_) and averted *P. vivax* clinical cases for each simulation. Estimates are further detailed in the Supplementary Materials. R version 4.1.3 was used for all calculations and to generate figures.

### Role of the funding agency

The funders had no role in study design, data collection and analysis, decision to publish, or preparation of the manuscript.

## Results

### Clinical case management versus mass campaign interventions

We compare the population-level impact of baseline CM (S_0_), strengthened CM (S_1_), a single round of *Pv*SeroTAT (S_2_), and a single round of MDA (S_4_) deployed during the low transmission season. Results are presented for three archetype Brazilian settings with varying levels of baseline transmission intensity and occupational exposure to malaria in the following order: an occupational exposure archetype setting in Itaituba, Pará with 23 cases per 1000 population; a peri-urban mixed archetype setting in Manaus, Amazonas with 114 cases per 1000; and a peri-domestic exposure archetype setting in São Gabriel da Cachoeira, Amazonas with 267 cases per 1000.

**Fig. 1:**
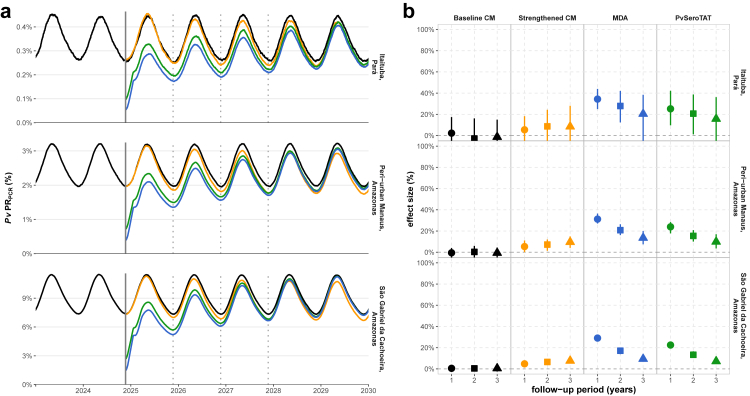
Impact of high efficacy primaquine deployed through strengthened CM, a single round of *Pv*SeroTAT, or MDA interventions in three archetype settings: Itaituba, Pará (23 cases per 1000); peri-urban Manaus, Amazonas (114 cases per 1000); and São Gabriel da Cachoeira, Amazonas (267 cases per 1000). **(a)** Model predicted *Pv*PR_PCR_ over time with interventions deployed at the end of 2024 (solid vertical grey line). Evaluation of the effect size compares the percent change in point *Pv*PR_PCR_ before the intervention and the point *Pv*PR_PCR_ one, two, and three years post-intervention (indicated by dotted vertical lines). *Pv*PR_PCR_ over time is shown for the baseline scenario (black), strengthened CM (yellow), MDA (blue), and *Pv*SeroTAT (green). **(b)** Mean point *Pv*PR_PCR_ reduction effect size (%) and 95% uncertainty intervals for 100 stochastic simulations per scenario evaluated at one, two, and three years follow-up. Values are reported in Supplementary Table S1.

S_0_ has no predicted change in transmission over time as the model is at an equilibrium. The impact of S_1_ increases over time due to introduction of high efficacy primaquine and G6PD testing. Across the three settings shown in Fig. 1, a mean point *Pv*PR_PCR_ reduction of 5.4% [95% UI: −10.7% to 18.3%], 5.3% [95% UI: 0.1%–11.2%], and 4.8% [95% UI: 2.2%–7.4%] is predicted at 12 months which increases to 8.7% [95% UI: −8.9% to 24.3%], 7.2% [95% UI: 1.4%–12%], and 6.6% [95% UI: 3.8%–8.8%] at 24 months, and to 8.4% [95% UI: −8.2% to 28.1%], 9.6% [95% UI: 4%–14.9%], and 7.6% [95% UI: 4.9%–10.2%] at 36 months (Supplementary Table S1). After several years, transmission reaches a new equilibrium due to the permanent improved case management that initially reduces prevalence until a new steady state is reached.

S_2_, a single *Pv*SeroTAT round with 80% coverage deployed in the low transmission season, is predicted to achieve 25.2% [95% UI: 9.6%–42.2%], 24% [95% UI: 17.8%–28.4%], and 22.5% [95% UI: 20.2%–24.8%] mean *Pv*PR_PCR_ reduction at 12 months in archetype settings (Fig. 1, Supplementary Table S1). S_2_ is predicted to have higher impact than strengthened CM but less impact than MDA due to imperfect sensitivity (assumed at 80% in this scenario). S_2_ mean *Pv*PR_PCR_ reduction wanes over time and reduces to 15.7% [95% UI −7.3% to 36.2%], 9.9% [95% UI: 3.6%–16.9%], and 7.2% [95% UI: 4.6%–9.8%] at 36 months compared to baseline prevalence before the campaign. A single MDA round (S_4_) is predicted to reach higher levels of prevalence reduction within the first 12 months as compared to S_1_ or S_2_ with a predicted 12-month mean *Pv*PR_PCR_ reduction of 34.4% [95% UI: 24.9%–44%], 31.2% [95% UI: 27.1%–36.4%], and 29.1% [95% UI: 26.8%–31.5%] (Supplementary Table S1); however, the benefits of the MDA decrease over time and transmission rebounds to baseline levels within five or more years depending on the setting.

Diagnostic performance of *Pv*SeroTAT campaigns and timing of deployment will vary predicted impact. Compared to S_2_ with 80% sensitivity and 80% specificity, improved sensitivity (S_2b_) versus improved specificity (S_2a_) of known diagnostic targets is predicted to have greater impact on population-level outcomes (Supplementary Fig. S1). S_2b_ with 90% sensitivity and 60% specificity is predicted reduce point *Pv*PR_PCR_ by 29.7% [95% UI: 18.2%–43.2%], 27.9% [95% UI: 22.2%–32.7%], and 25.7% [95% UI: 23.5%–27.7%] at 12 months follow-up (Supplementary Table S2). Deploying *Pv*SeroTAT during the low transmission period (S_2_) is predicted to achieve the highest point *Pv*PR_PCR_ reduction (Supplementary Fig. S2 and Table S2). For example, delaying deployment by six months during the period between low and high transmission (S_5a_) achieves less impact by reducing mean point *Pv*PR_PCR_ to 21.8% [95% UI: 6.5%–34.1%], 18.9% [95% UI: 14.6%–22.3%], and 17.2% [95% UI: 15.4%–19.5%] at 12 months.

For all interventions, the relative reduction in clinical cases over a 12-month period is lower than the relative reduction in point *Pv*PR_PCR_; however, the overall impact of different interventions follow the same trends. A greater effect size is predicted in low prevalence settings, although a larger number of cases are averted in high prevalence settings (Supplementary Tables S3 and S4). In the example of a single round of *Pv*SeroTAT, the mean *Pv*PR_PCR_ reductions predicted range from 25.2% [95% UI: 9.6%–42.2%], 24% [95% UI: 17.8%–28.4%], to 22.5% [95% UI: 20.2%–24.8%], while the mean proportion of clinical cases averted range from 20.7% [95% UI: 0.5%–36.5%], 18.3% [95% UI: 4.5%–32.2%], to 15.3% [95% UI: 2%–29.6%] in archetype settings (Supplementary Table S3). While Itaituba, which has lower baseline transmission, is predicted to achieve 20.7% [95% UI: 0.5%–36.5%] mean reduction in cases resulting in an average 900 [95% UI: 0–1500] cases per 100,000 averted, São Gabriel da Cachoeira with high transmission achieves 15.3% [95% UI: 2%–29.6%] mean relative reduction in cases and 7000 [95% UI: 900–13,900] averted cases per 100,000 (Supplementary Table S4).

### Multiple rounds

**Fig. 2:**
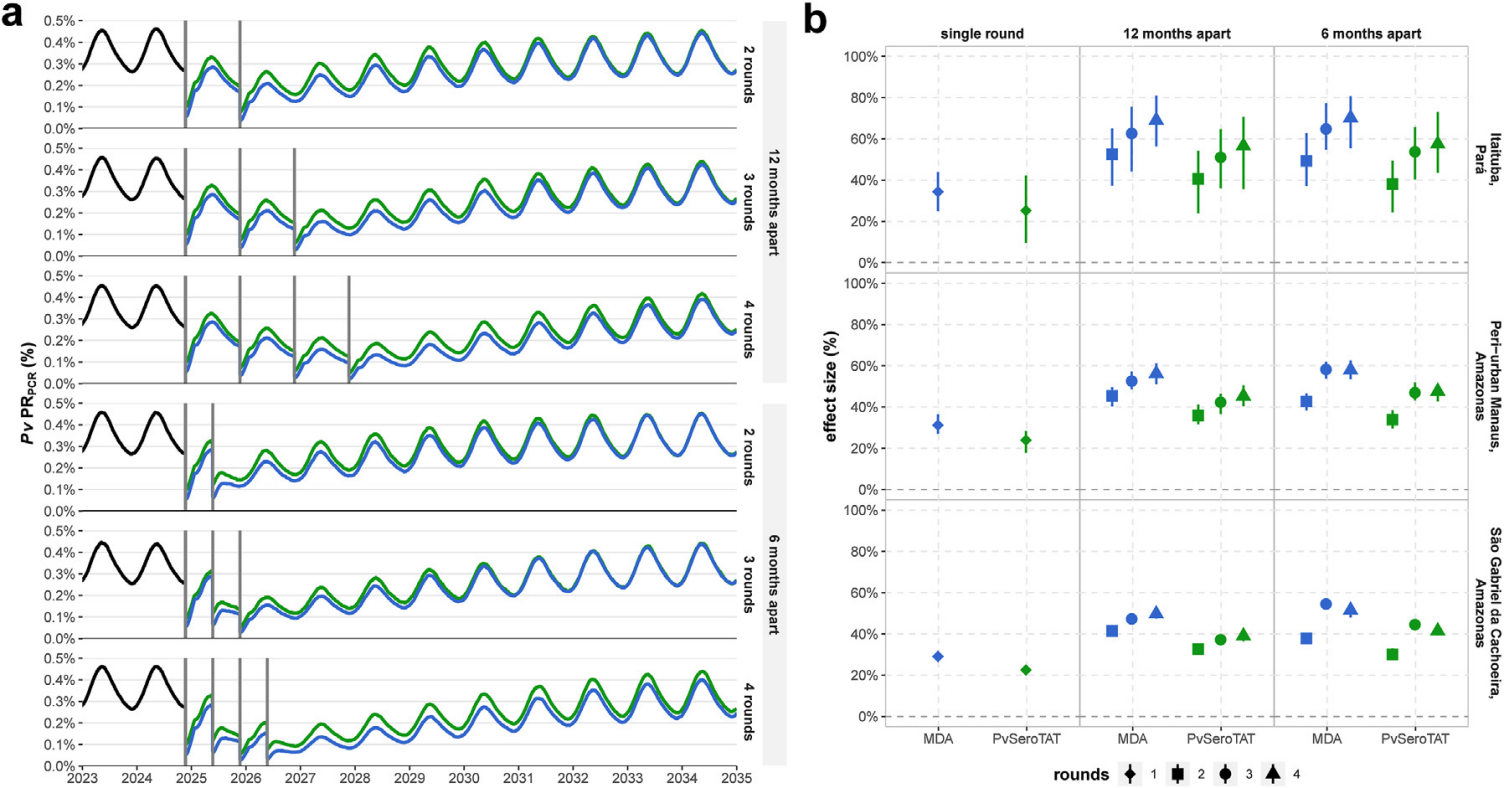
Impact of multiple rounds of PvSeroTAT and MDA interventions with variable timing between rounds. **(a)** PvPRPCR over time is shown for different deployment strategies in Itaituba, Pará. Each round is shown as a grey solid vertical line. Impact of PvSeroTAT on PvPRPCR is shown in green and MDA in blue. **(b)** Mean point PvPRPCR reduction effect size (%) and 95% uncertainty intervals (UI) for 100 stochastic simulations per scenario evaluated 12 months after the last round during follow-up. Values are reported in Supplementary Table S2.

We evaluated the impact of multiple rounds (two, three, or four) of *Pv*SeroTAT and MDA with variable timing between rounds (six months versus 12 months apart). Overall, high frequency strategies that increase the number of rounds and deploy rounds six months apart have higher population-level impact (Fig. 2, Supplementary Table S2). Rounds six months apart are more impactful than 12 months apart even if the second or fourth round are deployed during the high season. The highest impact for *Pv*SeroTAT campaigns is observed for scenarios with four rounds six months apart resulting in a predicted 57.5% [95% UI: 43.6%–73.1%], 47.6% [95% UI: 42.7%–51.2%], and 41.4% [95% UI: 39.2%–43.7%] reduction in point *Pv*PR_PCR_ at 12 months across archetype settings (Fig. 2, Supplementary Table S2).

Each additional mass campaign round increases mean point *Pv*PR_PCR_ reduction. In the example of Itaituba, Pará, multiple rounds of *Pv*SeroTAT 12 months apart with 12-month follow-up after the last round are predicted to reduce *Pv*PR_PCR_ by: 25.2% [95% UI: 9.6%–42.2%] for a single round, 40.6% [95% UI: 24%–54.1%] for two rounds, 51% [95% UI: 36%–64.7%] for three rounds, and 56.6% [95% UI: 35.7%–70.8%] for four rounds. In the same setting, we predict higher *Pv*PR_PCR_ reduction for deployment intervals six months apart: 38.1% [95% UI: 24.4%–49.4%] for two rounds, 53.7% [95% UI: 40.4%–65.8%] for three rounds, and 57.5% [95% UI: 43.6%–73.1%] for four rounds (Fig. 2, Supplementary Table S2). In the same setting, MDA has higher impact overall compared to *Pv*SeroTAT. Four rounds of MDA reduce mean point *Pv*PR_PCR_ by 69% [95% UI: 56.4%–80.9%] when deployed 12 months apart and by 70.2% [95% UI: 55.5%–80.6%] when deployed six months apart (Supplementary Table S2). Each additional round increases the relative reduction in clinical cases and number of cases averted per 100,000 (Supplementary Tables S3 and S4). For Itaituba, four rounds of *Pv*SeroTAT six months apart reduce clinical cases by 50.7% [95% UI: 26%–71.2%] at 12 months follow-up translating to 2100 [95% UI: 1100–3000] cases averted per 100,000. We observe similar trends for peri-urban Manaus and São Gabriel da Cachoeira settings (See Supplementary Materials).

### Treatment courses and G6PD tests

**Fig. 3:**
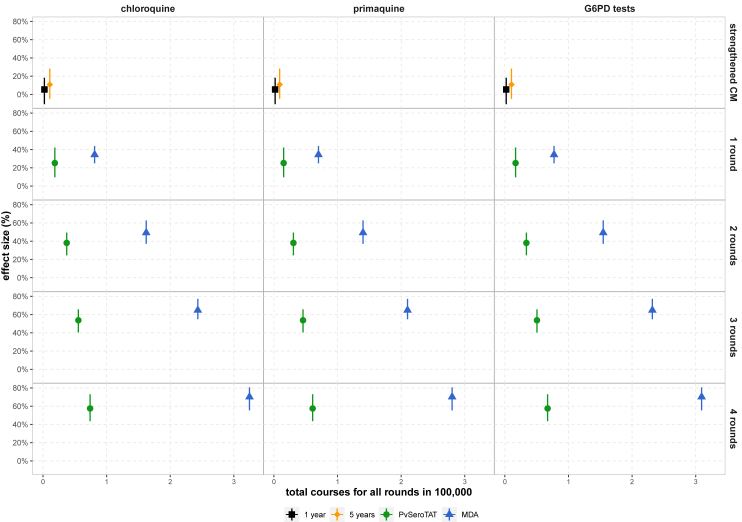
Total treatment courses and G6PD tests per 100,000 for scenarios by mean *Pv*PR_PCR_ effect size at 12 months in Itaituba, Pará. For strengthened CM, we show the cumulative number of courses and tests one or five years after deployment and the effect size at year one or year five respectively. We show results for multiple rounds of *Pv*SeroTAT and MDA interventions six months apart for two, three, or four rounds and Mean point *Pv*PR_PCR_ reduction effect size (%) at one-year follow-up. No significant difference in the total number of courses or tests is observed if rounds are six months or 12 months apart. Each scenario was modelled with a population size of 100,000 individuals.

We evaluated the required radical cure courses and G6PD tests required to achieve population-level impact for the various interventions (Supplementary Table S5). A single mass campaign with *Pv*SeroTAT or MDA requires more resources than five years of strengthened CM (Fig. 3). In Itaituba, a single round of *Pv*SeroTAT administers 0.15 per 100,000 or 15,300 [95% UI: 15,200–15,300] radical cure courses, a single MDA administers 70,000 [95% UI: 70,000–70,000] radical cure courses, and strengthened CM administers 9000 [95% UI: 8200–9800] courses over a five-year period (Fig. 3, Supplementary Table S5). *Pv*SeroTAT campaigns require significantly less resources than MDA campaigns. While a single MDA achieves an additional 9.2% reduction in point *Pv*PR_PCR_ compared to *Pv*SeroTAT (34.4% [95% UI: 24.9%–44%] versus 25.2% [95% UI: 9.6%–42.2%]) and averts an additional 300 cases (1200 [95% UI: 0–1500] vs. 900 [95% UI: 0–1500] cases per 100,000 averted) in Itaituba, it does so with 4.6 times the number of radical cure doses than *Pv*SeroTAT (Fig. 3, Supplementary Tables S4 and S5). Additionally, a single *Pv*SeroTAT campaign requires 4.6 times fewer G6PD tests than MDA (16,900 [95% UI: 16,800–16,900] tests versus 77,300 [95% UI: 77,300–77,300] tests). The gap between resource mobilisation for MDA as compared to *Pv*SeroTAT for small gains in impact are most apparent with four rounds of consecutive interventions. In Itaituba, four rounds of *Pv*SeroTAT deployed six months apart achieves 57.5% [95% UI: 43.6%–73.1%] *Pv*PR_PCR_ reduction with 60,900 [95% UI: 60,700–61,000] radical cure doses and 67,200 [95% UI: 67,100–67,300] G6PD tests compared to MDA administering 280,100 [95% UI: 280,000–280,200] courses and 309,300 [95% UI: 309,200–309,400] G6PD tests to reach 70.2% [95% UI: 55.5%–80.6%] *Pv*PR_PCR_ reduction.

For all rounds, MDA requires 4.6, 3.6, and 2.4 times more radical cure and G6PD tests than *Pv*SeroTAT in Itaituba, peri-urban Manaus, and São Gabriel da Cachoeira respectively. These results suggest that with decreasing transmission intensity, MDA campaigns require increasingly higher resources compared to *Pv*SeroTAT. For settings with high transmission intensity, since there are higher rates of infections, symptomatic or not, and as a result higher rates of seropositivity, we observe higher numbers of G6PD tests and treatments required for *Pv*SeroTAT in São Gabriel da Cachoeira (Supplementary Fig. S4) compared to peri-urban Manaus (Supplementary Fig. S3) and to Itaituba (Fig. 3).

### Layered interventions

**Fig. 4:**
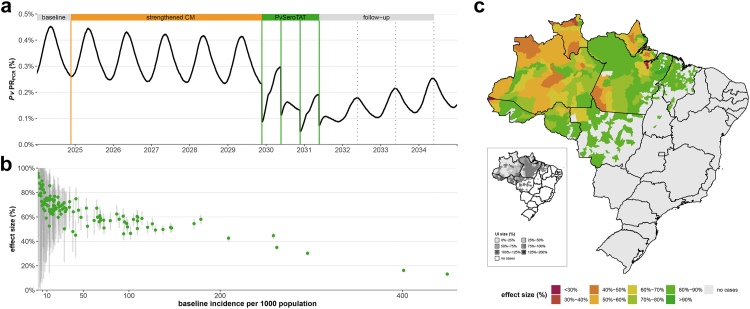
Impact of combined strengthened case management followed by four rounds of *Pv*SeroTAT mass campaigns deployed six months apart. **(a)***Pv*PR_PCR_ over time in Itaituba, Pará. Strengthened CM starts indefinitely at the end of 2024 (yellow vertical line). After five years, four rounds of *Pv*SeroTAT campaigns deployed six months apart (green vertical lines) are deployed. The effect size or relative change in point *Pv*PR_PCR_ is measured 12, 24, and 36 months after the last round (dotted vertical lines). **(b)** The mean point *Pv*PR_PCR_ reduction effect size (%) at 12 months (green point) and the 95% UI (grey vertical bars) are shown for all simulated settings (# municipalities = 126) by the baseline simulation incidence per 1000 population. **(c)** Each setting's mean point *Pv*PR_PCR_ reduction effect size (%) at 12 months is mapped by colour in the main plot and the size of the 95% UI is shown in the inset plot. For the simulated settings (n = 126), estimates of mean effect size are from model simulations. For non-simulated settings, we assumed a mean effect size from the simulated settings with a similar 2018 baseline incidence: 295 settings had an incidence of less than five per 1000 population which corresponded to a mean effect size of 83.6% [95% UI: 70.6%–94.9%] at 12 months in the simulated settings; two settings had an incidence between five and 10 cases per 1000 population which corresponded to a mean point prevalence effect size of 74.1% [61.3%–86.3%] at 12 months in the simulated settings; one setting had an incidence between 10 and 20 cases per 1000 population which corresponded to a mean point prevalence effect size of 69.5% [95% UI: 59.5%–79.4%] at 12 months in the simulated settings (Supplementary Table S6).

*Pv*SeroTAT interventions could be implemented together with strengthened CM to target both clinical cases seeking care and those with asymptomatic infections. We consider a potential scenario where the Brazilian health system has strengthened CM for five years followed by two (S_6a_) or four (S_6b_) rounds of *Pv*SeroTAT six months apart. Overall, we observe higher predicted impact of S_6b_ as compared to S_6a_ and S_3e_ (Fig. 4, Supplementary Table S2, Supplementary Fig. S5). Considering Itaituba for example, a mean point *Pv*PR_PCR_ reduction of 43.3% [95% UI: 25%–58.2%] is predicted for S_6a_ and a 59.7% [95% UI: 38.6%–74.1%] reduction for S_6b_ (Fig. 4a) compared to a reduction of 38.1% [95% UI: 24.4%–49.4%] for S_3c_ and 57.5% [95% UI: 43.6%–73.1%] for S_3e_ without strengthened CM. In this setting, strengthened CM alone compared to the same 12-month evaluation period achieves 11.3% [95% UI: −7.9% to 31.7%] point *Pv*PR_PCR_ reduction compared to S_6a_'s evaluation period and 12% [95% UI: −11.5% to 28.3%] point *Pv*PR_PCR_ reduction compared to S_6b_'s evaluation period.

Across all Brazilian *P. vivax* endemic settings, we predict a wide range of impact estimates due to heterogenous baseline transmission levels (Fig. 4b, Supplementary Table S6). For S_6b_, the lowest impact predicted is 13.2% [95% UI: 11.9%–14.5%] point *Pv*PR_PCR_ reduction at 12 months in Oeiras do Pará, Pará, which has the highest baseline incidence of 449 cases per 1000. For the same scenario, the highest impact predicted is 95.7% [95% UI: 43.3%–100%] in Paragominas, Pará, which has a baseline incidence of 0.3 cases per 1000 population.

The highest transmission settings with over 200 cases per 1000 are predicted to achieve the lowest impact: a mean of 23.5% [95% UI: 11.8%–33.2%] *Pv*PR_PCR_ reduction for S_6a_ and a mean of 30.3% [95% UI: 13.6%–44.4%] *Pv*PR_PCR_ reduction for S_6b_ (Supplementary Table S6). Three years following the last *Pv*SeroTAT campaign, transmission rebounds in both scenarios; however, there is some continued effect. At 36 months, there is a predicted mean 8.4% [95% UI: 2.5%–14.1%] point *Pv*PR_PCR_ reduction maintained compared to baseline levels before the interventions for S_6a_ and a mean 9.9% [95% UI: 2.5%–17.3%] point *Pv*PR_PCR_ reduction for S_6b_ (Supplementary Table S6).

Low and moderate transmission settings benefit most from both intervention strategies. For example, settings with an incidence between 10 and 20 cases per 1000 are predicted to reduce point *Pv*PR_PCR_ at 12 months by a mean of 69.5% [95% UI: 59.5%–79.4%] with S_6b_ (Fig. 4, Supplementary Table S6). For the same scenario, the lowest transmission settings with a baseline incidence of less than five cases per 1,000, are predicted to reduce point *Pv*PR_PCR_ by a mean of 83.6% [95% UI: 70.6%–94.9%]. Assuming transmission dynamics are similar in non-modelled settings with very low incidence (less than 100 cases reported in 2018, n = 295), the majority of Brazilian settings could potentially reach at least 80% reduction in point *Pv*PR_PCR_ reduction at 12 months if this novel combined strategy is implemented at high coverage across the endemic region (Fig. 4c, Supplementary Table S6).

However, for low transmission settings, the uncertainty in model predictions is also very high (Fig. 4b–c, Supplementary Fig. S6). Settings with a baseline incidence of less than five cases per 1000 compared to settings with five to 10 cases per 1000 have wider uncertainty intervals and higher rates of simulation fadeout (Supplementary Fig. S6). Additionally, while not included in mean impact estimates, many simulations in low transmission settings reach zero cases before interventions are introduced. For example, in Paragominas, Pará, 57 and 47 out of the 100 stochastic simulations have zero cases before interventions S_6a_ and S_6b_ are deployed respectively. Out of the simulations with transmission, downward trends towards fadeout are present which result in 80% of simulations with no malaria 12 months after the last *Pv*SeroTAT campaign. Therefore, careful interpretation of impact estimates is required for such settings.

## Discussion

Interventions that directly target the *P. vivax* hypnozoite reservoir for radical cure treatment and prevent transmission from asymptomatic primary infections or relapses are essential for accelerating malaria elimination efforts. A serological diagnostic tool has been developed and validated to detect recent *P. vivax* infections and potential carriers of hypnozoites with 80% sensitivity and 80% specificity. Such a diagnostic has the potential to screen asymptomatic carriers of hypnozoites for targeted radical cure treatment through mass campaign interventions called *Pv*SeroTAT. Our model predictions show that strengthened case management with *Pv*SeroTAT campaigns with efficacious radical cure treatment at high coverage have the potential to reduce point *Pv*PR_PCR_ by a mean 74.1% [95% UI: 61.3%–86.3%] or more in low transmission settings with less than 10 cases per 1000 population if deployed for several rounds at short intervals across the Brazilian Amazon Region.

Brazil's strong case management practices have helped to significantly reduce malaria over the last 20 years; nevertheless, Brazil has yet to eliminate *P. vivax*. While the national malaria program plans to further strengthen management of symptomatic clinical cases of *P. vivax* by introducing single dose tafenoquine*,* the already comparatively high rates of effective radical cure limit the effectiveness of improved CM, also supported by our results modelling higher efficacy and higher adherence of radical cure with primaquine.^12^ Additional interventions targeting the asymptomatic reservoir are thus needed if Brazil is to achieve elimination goals expeditiously.

Mass campaigns that can reach and treat asymptomatic cases with radical cure at high coverage can have substantial impact on *P. vivax* malaria. Our modelling results show that a single mass campaign with MDA or with *Pv*SeroTAT screening could achieve between 20% and 35% prevalence reduction at 12-month follow-up with 80% coverage. MDA campaigns would achieve the greatest impact because all carriers of parasites who are covered by the campaign will receive radical cure treatment. However, MDA is resource intensive – all individuals need G6PD screening and radical cure treatment leading to high rates of overtreatment. For *Pv*SeroTAT, while imperfect performance of the serological diagnostic will screen out false positive individuals resulting in slightly lower impact than MDA, only seropositive individuals will be screened for G6PD testing for radical cure eligibility resulting in significant less overtreatment. Depending on the setting, *Pv*SeroTAT administers 4.6 to 2.4 times less treatment and G6PD tests compared to MDA. This rate is highest in low transmission settings where mass campaigns have the greatest impact. Given the lower number of G6PD tests and treatments required, *Pv*SeroTAT could potentially be less costly to implement than MDA if the total cost of serological testing is less than the additional G6PD testing and treatment for MDA. This is particularly true if directly observed treatment is required for optimal PQ radical cure efficacy.^30^ G6PD testing has been shown to be cost-effective in preventing primaquine-associated hospitalisations and is likely to be required for such campaigns.^10^ A formal cost-impact analysis will be required to confirm the cost-benefit of *Pv*SeroTAT.

The impact of *Pv*SeroTAT can be increased by deploying the intervention in the low transmission season and by increasing the number of campaign rounds delivered, albeit with diminishing returns. Impact can be further increased by shortening the interval between campaign rounds because shorter periods provide less time for transmission to rebound. For examples, four rounds of *Pv*SeroTAT six months apart are predicted to achieve 82% of the impact of MDA with 4.6 fewer radical cure treatments in a low transmission setting such as Itaituba in the state of Pará. The optimal deployment strategy will however depend on impact targets, cost-benefit, and operational feasibility of delivering multiple rounds of a serological diagnostic testing, G6PD testing, and radical cure administration over a specific time frame. As for other mass screen and treat interventions, operational feasibility may be improved by deploying targeted *Pv*SeroTAT in residual transmission pockets or in high-risk populations (e.g. minors, forest workers, etc.). Like MDA, *Pv*SeroTAT will rarely lead to interruption of local transmission alone, but it can accelerate the path to elimination by potentially achieving 74% reduction or more in *Pv*PR_PCR_ in low transmission settings with a campaign achieving high coverage and adherence. While our model predictions in settings with less than five cases per 1000 population have a high degree of uncertainty due to the strong stochastic effect of modelling low transmission and rare infection events, our results indicate a strong potential for the majority of settings in Brazil to reach pre-elimination phases quickly if frequent campaigns and high coverage are achieved. Such campaigns alone may not achieve elimination; however, they can significantly reduce the malaria burden in a short period which can allow programs to transition to implementation of reactive case management and other strategies to prevent rebound.

Brazil is an ideal setting to model the introduction of *Pv*SeroTAT due to several reasons: there is a centralised health care system with high case management coverage of clinical cases and free radical cure treatment; recent approval of tafenoquine by local authorities and pilot study of G6PD quantitative diagnostic testing before the use of tafenoquine has shown promising results (Lacerda M, Fundação de Medicina Tropical Dr. Heitor Vieira Dourado, Brazil; personal communication), allowing Brazil to consider different radical cure regimens; and Brazil has a heterogeneous transmission landscape from very high to pre-elimination settings and peri-urban to occupational malaria exposure settings. In this context, introduction of *Pv*SeroTAT provides a much greater impact on *P. vivax* transmission than further strengthening case management. This is likely to be quite different in other countries, particularly for most of the Asia–Pacific, where rates of effective radical cure for clinical infections are much lower.^15^ In such settings, *Pv*SeroTAT should be either implemented after CM is strengthen or go hand-in-hand with CM strengthening.

Our modelling results are encouraging, suggesting that introducing serology-based screening and mass drug campaigns, especially in a country like Brazil where currently case management and surveillance are prioritised, will rapidly reduce prevalence; however, further validation with clinical trial evidence is required. While a rapid serological diagnostic test is currently being developed to facilitate delivery, ongoing and future clinical trials will provide the strongest evidence for the range of prevalence and incidence reduction that can be achieved in real-world settings. While we model *Pv*SeroTAT as a population-wide mass campaign, the delivery methods, achievable population coverage, and use cases may change with new evidence. For example, *Pv*SeroTAT could be deployed focally in high transmission areas, to high-risk groups such as children, to forest workers in communities, or in hospital settings as a post-discharge screening tool for vulnerable patients. Once the use cases are better understood and more evidence is available, additional modelling studies can provide improved impact estimates.

Our modelling results summarise the potential trends in population-level impact of introducing novel interventions to combat *P. vivax*; however, model uncertainty and uncertainty in our parameterisation should be considered in the interpretation of our results. Mean relative reduction or cases averted are reported along with uncertainty intervals to show the wide range of model predictions. Our predictions should be considered as a summary statistic of a complex process. We show that in very low transmission settings, stochastic noise and fadeout result in more unstable transmission dynamics and greater variation between simulations. In some scenarios, we observe no malaria cases after mass campaigns; however, such elimination events are likely accelerated by model fadeout and should be interpreted with caution. Simulated settings with baseline incidence greater than five cases per 1000 did not fadeout and provide more reliable impact estimates. Another limitation of our work is that we also did not model imported malaria cases; therefore, local transmission dynamics in real-world communities are likely to differ compared to our model. Our baseline assumptions were calibrated to 2018 transmission levels before the Covid-19 pandemic and may not reflect the current and future trends of malaria in Brazil. Additionally, we assume homogenous mixing of populations in modelled settings, particularly in settings covering large regions of the Amazon Basin where pockets of heterogeneous transmission are masked. Radical cure efficacy is based on best estimates for Brazil, while they may be different in programmatic *Pv*SeroTAT implementation, particularly achievable intervention coverage. Finally, we did not model reductions in adherence rates per round or campaign coverage that could be observed in specific populations with potentially low treatment adherence or resistance to mass campaigns such as gold miners. Further studies are needed to better understand implementing novel strategies in less accepting or accessible populations.^26^^,^^31^

Nonetheless, our modelling study demonstrates the advantages of deploying *Pv*SeroTAT compared to MDA or strengthened CM alone. By comparing population-level impact predictions across different settings and quantifying required tests and treatment courses for a range of deployment strategies, our study shows the potential of *Pv*SeroTAT to accelerate malaria elimination efforts. *Pv*SeroTAT mass campaigns deployed at high coverage and frequency along with strengthened case management have the potential to reduce point prevalence by 74% or more in low transmission settings. Such interventions should be considered for future clinical studies to validate *Pv*SeroTAT and for future implementation to accelerate elimination efforts.

## Contributors

NN, MTW, and IM and conceived the study and designed the methodology. NN and TO performed the modelling simulations. NN led the formal data analysis and generated the figures and tables. NN, MTW, and IM wrote the manuscript. TO and IM accessed and verified the data. MVGL and WMM were involved in previous parametrization and validation of the model. TO, WM, MVGL, MTW, and IM reviewed the results and manuscript. All authors approved the final draft of the manuscript.

## Data sharing statement

The model code is publicly available online at https://github.com/MWhite-InstitutPasteur/Pvivax_TQ_IBM. Restrictions apply for SIVEP malaria case reporting data. SIVEP data is owned by the Ministry of Health of Brazil which can be attained by meeting data access criteria by contacting the Health System Informatics Department (DATASUS) at datasus@saude.gov.br. Demographic data is publicly available at https://www.ibge.gov.br/. All other relevant data for modelling calibrations are publicly available in cited publications.

## Editor note

The Lancet Group takes a neutral position with respect to territorial claims in published maps and institutional affiliations.

## Declaration of interests

IM and MW declare a Patent for *P. vivax* serological markers of recent exposure and their applications in public health interventions (PCT/US17/67,926). The other authors have no completing interest to declare.
