# Supplementary material for: Accelerating towards P. vivax elimination with a novel serological test-and-treat strategy: a modelling case study in Brazil
Source: Lancet Reg Health Am. 2023 May 19;22:100511. doi: 10.1016/j.lana.2023.100511 (PMC10209700; doi:10.1016/j.lana.2023.100511)
Supplement: Supplementary Methods, Figs. S1–S6, and Tables S1–S6 [file mmc1.pdf]

Supplementary Materials

Accelerating towards *P. vivax* elimination with a novel serological test-and-treat strategy: a modelling case study in Brazil

Narimane Nekkab PhD[1,2,3], Thomas Obadia PhD[1,4], Wuelton M Monteiro PhD[5,6], Marcus Lacerda MD[6,7], Michael White PhD[1], Ivo Mueller PhD[8,9]

[1] Institut Pasteur, Université Paris Cité, G5 Épidémiologie et Analyse des Maladies Infectieuses, Paris, France

[2] Swiss Tropical and Public Health Institute, Allschwil, Switzerland

[3] University of Basel, Basel, Switzerland

[4] Institut Pasteur, Université Paris Cité, Bioinformatics and Biostatistics Hub, Paris, France

[5] Escola Superior de Ciências da Saúde, Universidade do Estado do Amazonas, Manaus, Brazil

[6] Diretoria de Ensino e Pesquisa, Fundação de Medicina Tropical Dr. Heitor Vieira Dourado, Manaus, Brazil

[7] Instituto Leônidas e Maria Deane, Fundação Oswaldo Cruz, Manaus, Brazil

[8] Population Health & Immunity Division, Walter and Eliza Hall Institute of Medical Research, Parkville, Australia

[9] Department of Medical Biology, University of Melbourne, Melbourne, Australia

*Correspondence to:
narimane.nekkab@swisstph.ch
mueller@wehi.edu.au

Table of Contents

Methods **2**

Table S1. .... **4**

Figure S1. .... **5**

Table S2. .... **6**

Table S3. .... **7**

Figure S2. .... **8**

Table S4. .... **9**

Table S5. .... **10**

Table S6. .... **14**

Figure S5. .... **15**

Figure S6. .... **16**

Methods

Scenario modelling

For each setting modelled, the population represents a Brazilian municipality with calibrated transmission intensity estimates using 2018 *P. vivax* incidence per 1000 population reported in the Malaria Epidemiological Surveillance Information System (SIVEP-malaria). G6PD deficiency prevalence estimated were retrieved from cross-sectional surveys.[11,30] In the archetype settings, G6PDd prevalence amongst surveyed males in the archetype settings ranged from 0% to 3.3%. Across Brazil, G6PDd prevalence ranged from 0% to 16%. The average age of the population was estimated from demographic datasets reported by the Brazilian Institute of Geography and Statistics (IBGE). Additionally, using the age and gender distribution of cases, we determined two modes of exposure (peri-domestic or occupational) and calibrated mosquito bites accordingly.[11]

Assuming S_0 CM, twenty different mass campaign scenarios of MDA and PvSeroTAT with were simulated with G6PD testing and radical cure with high efficacy primaquine, 80% adherence, and 80% coverage (Table 1). For MDA, G6PD testing was administered to all individuals included in the intervention coverage. For *Pv*SeroTAT, G6PD testing was only conducted following positive serological diagnostic results after screening. Three of these scenarios account for varying sensitivity and specificity of the diagnostic tool (S_2, S_{2a}, S_{2b}). With the exception of S_{2a} and S_{2b}, all *Pv*SeroTAT scenarios assumed 80% sensitivity and 80% specificity of the diagnostic tool. With the exception of S_{5a} and S_{5b}, all other *Pv*SeroTAT scenarios were deployed during the low transmission season. S_{5a} simulated deployment between the low and high season ("mid-season") and S_{5b} deployment was during the high season. Scenarios S_{3a-f} considered two, three or four consecutive rounds with either a six-month or a 12-month interval between rounds. All single intervention scenarios simulated baseline CM to differentiate mass intervention and strengthened CM effects. The two country-wide combined intervention scenarios simulated strengthened CM for five years followed by two (S_{6a}) or four consecutive (S_{6b}) rounds of *Pv*SeroTAT six months apart as it assumed that in a programmatic implementation the same treatment would be used for *Pv*SeroTAT and CM. Scenarios S_4 and S_{4a-f} consider the same deployment strategies for MDA interventions as a comparator with 80% deployment coverage.

One hundred simulations were run for each setting and scenario for a duration of 20 years spanning from 2015 to 2035. S_{1-5} results are shown for the three archetype settings: Itaituba, an archetype of occupational exposure in Pará state with an incidence of 23 cases per 1,000 in 2018; São Gabriel da Cachoeira, an archetype of peri-domestic transmission in Amazonas state with an incidence of 267 cases per 1,000 in 2018; and peri-urban Manaus, a mixed-exposure archetype setting where transmission is significantly higher than in the municipality as a whole with 114 cases per 1,000 in 2018 (compared to the entire municipality with a population of over two million people mainly in urban non-endemic areas where the incidence is estimated to be 6 cases per 1000). S_{6a} and S_{6b} results are given for all municipalities that reported at least 100 *P. vivax* cases in 2018 (n=126). For 298 municipalities that reported less than 100 cases in 2018, we assumed an effect size category similar to the median effect size of modelled municipalities with the same incidence category.

Impact calculations

For each simulation, we estimated the effect size as the relative difference during the baseline versus the follow-up period. We calculated the effect size for point $PvPR_{PCR}$ and report the mean and uncertainty interval of 100 stochastic simulations of the same scenario. We also calculate the effect size of clinical cases by comparing the cumulative cases during the baseline period as compared to the follow-up period. The number of cases averted is the difference between the cumulative number of cases between these periods reported at cases per 100,000 population.

For example, baseline point *Pv*PR_{PCR} was measured one day before rollout of the intervention and was compared to the point *Pv*PR_{PCR} estimate one, two, and three years following the introduction of strengthened case management, a single mass campaign, or the last round of mass campaigns during the same time point in the transmission period. Since scenarios with two or four rounds six months apart (S_{3a}, S_{3e}, S_{4a}, S_{4e}, S_{6a}, S_{6b}) were last deployed during the high transmission period, we estimated the baseline point *Pv*PR_{PCR} estimate six months before the first round (during the high transmission period) and compared it to 12 months after the last round to match the seasonality.

Treatment courses were counted as a single course for a three-day CQ and seven-day PQ course. G6PD tests and *Pv*SeroTAT were calculated as a single test. For CM scenarios, the cumulative total number of courses and tests were calculated after one or five years. For mass campaigns, we summed the total number of treatment courses, G6PD tests and *Pv*SeroTAT for all rounds deployed.

For S_{6a}, S_{6b}, for municipalities reporting < 100 cases that we did not simulate, we assumed an average effect from simulated municipalities with very low transmission and the same baseline incidence range We evaluate impact on *Pv*PR_{PCR} at 12, 24, or 36 months after the second or fourth *Pv*SeroTAT round by comparing follow-up point *Pv*PR_{PCR} to baseline point *Pv*PR_{PCR} during the same high transmission period before introducing strengthened CM seven or eight years prior.

Table S1. *Pv*PR$_{PCR}$ effect size (%) with 95% uncertainty interval for single round interventions across archetype settings.

	Follow-up (months)	*Itaituba, Pará*	*Peri-urban Manaus, Amazonas*	*São Gabriel da Cachoeira, Amazonas*
Baseline (S_0)	12	2.3% [-9.7%-17.4%]	-0.5% [-8.5%-4%]	0.6% [-1.6%-3%]
	24	-2.8% [-15.7%-16.2%]	0.3% [-5.8%-5.9%]	0.5% [-1.7%-2.8%]
	36	-1.4% [-14%-15%]	-0.7% [-7%-4.5%]	0.5% [-2.5%-3%]
Strengthened CM (S_1)	12	5.4% [-10.7%-18.3%]	5.3% [0.1%-11.2%]	4.8% [2.2%-7.4%]
	24	8.7% [-8.9%-24.3%]	7.2% [1.4%-12%]	6.6% [3.8%-8.8%]
	36	8.4% [-8.2%-28.1%]	9.6% [4%-14.9%]	7.6% [4.9%-10.2%]
MDA (S_4)	12	34.4% [24.9%-44%]	31.2% [27.1%-36.4%]	29.1% [26.8%-31.5%]
	24	27.9% [12.4%-42.1%]	20.8% [16.1%-26.4%]	17.1% [13.9%-19.8%]
	36	20.4% [-5.2%-38.5%]	13.5% [7.5%-20%]	9.4% [6.8%-12.3%]
PvSeroTAT (S_2)	12	25.2% [9.6%-42.2%]	24% [17.8%-28.4%]	22.5% [20.2%-24.8%]
	24	20.7% [1.2%-38.7%]	15.4% [10%-20.8%]	13.3% [10.7%-16.6%]
	36	15.7% [-7.3%-36.2%]	9.9% [3.6%-16.9%]	7.2% [4.6%-9.8%]

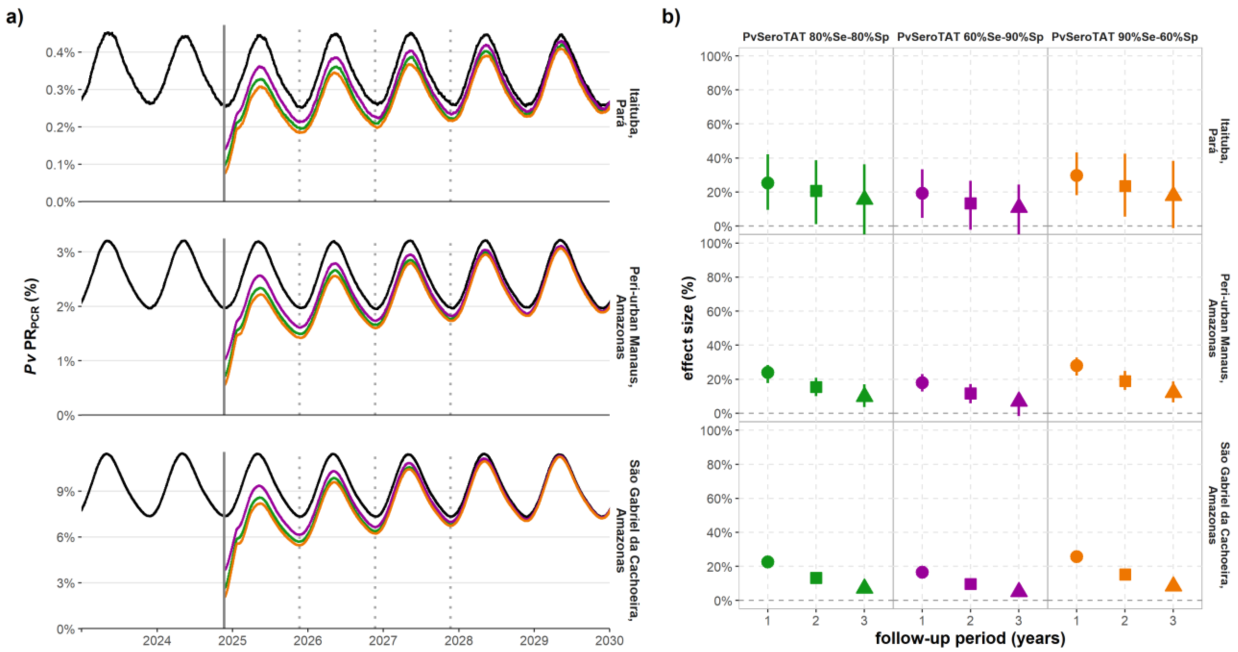

Figure S1. Impact of varying sensitivity and specificity of *Pv*SeroTAT with a single round. **(A)** Model predicted *Pv*PR$_{PCR}$ over time with interventions deployed at the end of 2024 (solid vertical grey line). Evaluation of the effect size compares the percent change in point *Pv*PR$_{PCR}$ before the intervention and the point *Pv*PR$_{PCR}$ one, two, and three years post-intervention (indicated by dotted vertical lines). *Pv*PR$_{PCR}$ over time is shown for the baseline scenario S_0 (black), S_2 PvSeroTAT (green) with 80% sensitivity and 80% specificity, S_{2a} *Pv*SeroTAT (purple) with 60% sensitivity and 90% specificity, S_{2b} PvSeroTAT (orange) with 90% sensitivity and 60% specificity. **(B)** Mean effect size (%) and 95% UI of change in point *Pv*PR$_{PCR}$ for 100 stochastic simulations per scenario evaluated at one, two, and three-years follow-up.

Table S2. Mean *Pv*PR_{PCR} effect size (%) and 95% uncertainty interval of 100 stochastic simulations of scenarios across archetype settings over 12 months follow-up.

	Intervention	*Rounds*	*Interval between rounds (months)*	*Itaituba, Pará*	*Peri-urban Manaus, Amazonas*	*São Gabriel da Cachoeira, Amazonas*
S_0	Baseline CM			2.3% [-9.7%-17.4%]	-0.5% [-8.5%-4%]	0.6% [-1.6%-3%]
S_1	Strengthened CM			5.4% [-10.7%-18.3%]	5.3% [0.1%-11.2%]	4.8% [2.2%-7.4%]
S_2	*Pv*SeroTAT	1		25.2% [9.6%-42.2%]	24% [17.8%-28.4%]	22.5% [20.2%-24.8%]
S_{2a}	*Pv*SeroTAT	1		19.2% [4.7%-33.4%]	17.9% [12.7%-23.1%]	16.5% [13.8%-18.7%]
S_{2b}	*Pv*SeroTAT	1		29.7% [18.2%-43.2%]	27.9% [22.2%-32.7%]	25.7% [23.5%-27.7%]
S_{3a}	*Pv*SeroTAT	2	6	38.1% [24.4%-49.4%]	33.9% [29.7%-38.4%]	30% [27.6%-32.9%]
S_{3b}	*Pv*SeroTAT	2	12	40.6% [24%-54.1%]	35.9% [31.6%-41.2%]	32.6% [30.5%-34.7%]
S_{3c}	*Pv*SeroTAT	3	6	53.7% [40.4%-65.8%]	46.9% [43.3%-51.9%]	44.5% [42.3%-46.2%]
S_{3d}	*Pv*SeroTAT	3	12	51% [36%-64.7%]	42.3% [36.6%-46.5%]	37.2% [35.1%-39.4%]
S_{3e}	*Pv*SeroTAT	4	6	57.5% [43.6%-73.1%]	47.6% [42.7%-51.2%]	41.4% [39.2%-43.7%]
S_{3f}	*Pv*SeroTAT	4	12	56.6% [35.7%-70.8%]	45.3% [40.4%-50.5%]	39% [36.4%-41.6%]
S_4	MDA	1		34.4% [24.9%-44%]	31.2% [27.1%-36.4%]	29.1% [26.8%-31.5%]
S_{4a}	MDA	2	6	49.3% [37.1%-62.8%]	42.7% [38.4%-46.6%]	37.9% [35.7%-40%]
S_{4b}	MDA	2	12	52.5% [37.3%-64.9%]	45.4% [40.3%-49.5%]	41.5% [39.6%-43.6%]
S_{4c}	MDA	3	6	64.8% [54.9%-77.2%]	58.2% [53.8%-61.9%]	54.5% [52.6%-56.4%]
S_{4d}	MDA	3	12	62.6% [44.1%-75.6%]	52.6% [48.6%-57.2%]	47.3% [44.9%-49.6%]
S_{4e}	MDA	4	6	70.2% [55.5%-80.6%]	57.9% [53.5%-62.6%]	51.5% [48%-53.8%]
S_{4f}	MDA	4	12	69% [56.4%-80.9%]	56.2% [51%-61.3%]	49.7% [47.4%-52%]
S_{5a}	*Pv*SeroTAT mid-season	1		21.8% [6.5%-34.1%]	18.9% [14.6%-22.3%]	17.2% [15.4%-19.5%]
S_{5b}	*Pv*SeroTAT high season	1		22.8% [9.8%-35.2%]	21% [17%-24.6%]	18.6% [16.6%-20.5%]
S_{6a}	$S_1 + S_{3a}$	5 years + 2 rounds	6	43.3% [25%-58.2%]	38% [33.2%-43%]	33.4% [31.4%-35.6%]
S_{6b}	$S_1 + S^{3e}$	5 years + 4 rounds	6	59.7% [38.6%-74.1%]	50.5% [45%-55.7%]	44.7% [42%-47.3%]

Table S3. Mean clinical cases effect size (%) and 95% uncertainty interval of 100 stochastic simulations of scenarios across archetype settings over 12 months follow-up.

	Intervention	*Rounds*	*Interval between rounds (months)*	*Itaituba, Pará*	*Peri-urban Manaus, Amazonas*	*São Gabriel da Cachoeira, Amazonas*
S_0	Baseline CM			-2.2%<br>[-19.4%-12.8%]	-0.9%<br>[-6.6%-3.9%]	-0.3%<br>[-3%-2.2%]
S_1	Strengthened CM			6.7%<br>[-9.6%-23.7%]	8.5%<br>[2.4%-15.5%]	8.4%<br>[3.5%-12.6%]
S_2	*Pv*SeroTAT	1		20.7%<br>[0.5%-36.5%]	18.3%<br>[4.5%-32.2%]	15.3%<br>[2%-29.6%]
S_{2a}	*Pv*SeroTAT	1		15.2%<br>[-3.5%-30.4%]	13.6%<br>[3.2%-23.6%]	11.2%<br>[0.4%-23.1%]
S_{2b}	*Pv*SeroTAT	1		24.4%<br>[2.3%-42.9%]	21.8%<br>[6.9%-35.6%]	17.9%<br>[3.7%-33.3%]
S_{3a}	*Pv*SeroTAT	2	6	32.7%<br>[8.9%-49.2%]	28.7%<br>[10.4%-45%]	22.6%<br>[4.8%-41.9%]
S_{3b}	*Pv*SeroTAT	2	12	33.8%<br>[1.8%-54%]	27.2%<br>[7.9%-44.8%]	20.3%<br>[2.9%-39.1%]
S_{3c}	*Pv*SeroTAT	3	6	46.4%<br>[22.2%-70.8%]	36.2%<br>[16.3%-56.1%]	28.4%<br>[6.7%-51.7%]
S_{3d}	*Pv*SeroTAT	3	12	40.8%<br>[11.2%-63.6%]	30.5%<br>[9.6%-49.6%]	21.2%<br>[1.3%-42.3%]
S_{3e}	*Pv*SeroTAT	4	6	50.7%<br>[26%-71.2%]	38.8%<br>[17%-59.9%]	29.1%<br>[5.4%-53.9%]
S_{3f}	*Pv*SeroTAT	4	12	46%<br>[16.8%-67.9%]	31.3%<br>[9.9%-52.9%]	19.9%<br>[-0.9%-43.1%]
S_4	MDA	1		28.5%<br>[2.1%-45.5%]	24.7%<br>[8.9%-40.7%]	20.3%<br>[4.2%-38%]
S_{4a}	MDA	2	6	43.3%<br>[12.2%-61.2%]	36.5%<br>[16%-56.2%]	29.2%<br>[7.9%-51.9%]
S_{4b}	MDA	2	12	44.2%<br>[16.8%-65.1%]	35.4%<br>[14.5%-55.3%]	26.8%<br>[6.1%-49.5%]
S_{4c}	MDA	3	6	56.6%<br>[29.5%-73.8%]	45.9%<br>[21.8%-67.8%]	36.5%<br>[10.5%-62.5%]
S_{4d}	MDA	3	12	52.7%<br>[20.7%-75.4%]	39.3%<br>[15.6%-61.7%]	28.4%<br>[4%-54.2%]
S_{4e}	MDA	4	6	63.7%<br>[32.9%-81%]	48.4%<br>[23.4%-71%]	37%<br>[8.5%-65.1%]
S_{4f}	MDA	4	12	58.3%<br>[27.1%-79.4%]	40.3%<br>[14.4%-64.4%]	26.9%<br>[-0.2%-55%]
S_{5a}	*Pv*SeroTAT mid-season	1		17.3%<br>[-2.2%-32.3%]	15.4%<br>[4%-26.1%]	13%<br>[1.8%-24.9%]
S_{5b}	*Pv*SeroTAT high season	1		19.6%<br>[2.5%-35.2%]	17.9%<br>[4.8%-29.2%]	14.8%<br>[2.1%-28.1%]
S_{6a}	$S_1 + S_{3a}$	5 years + 2 rounds	6	40.4%<br>[12.8%-58.4%]	35.1%<br>[19.6%-50%]	28.6%<br>[12.9%-45.7%]
S_{6b}	$S_1 + S^{3e}$	5 years + 4 rounds	6	55%<br>[29.9%-73.8%]	44.1%<br>[22.9%-62.7%]	34.5%<br>[13.1%-56.9%]

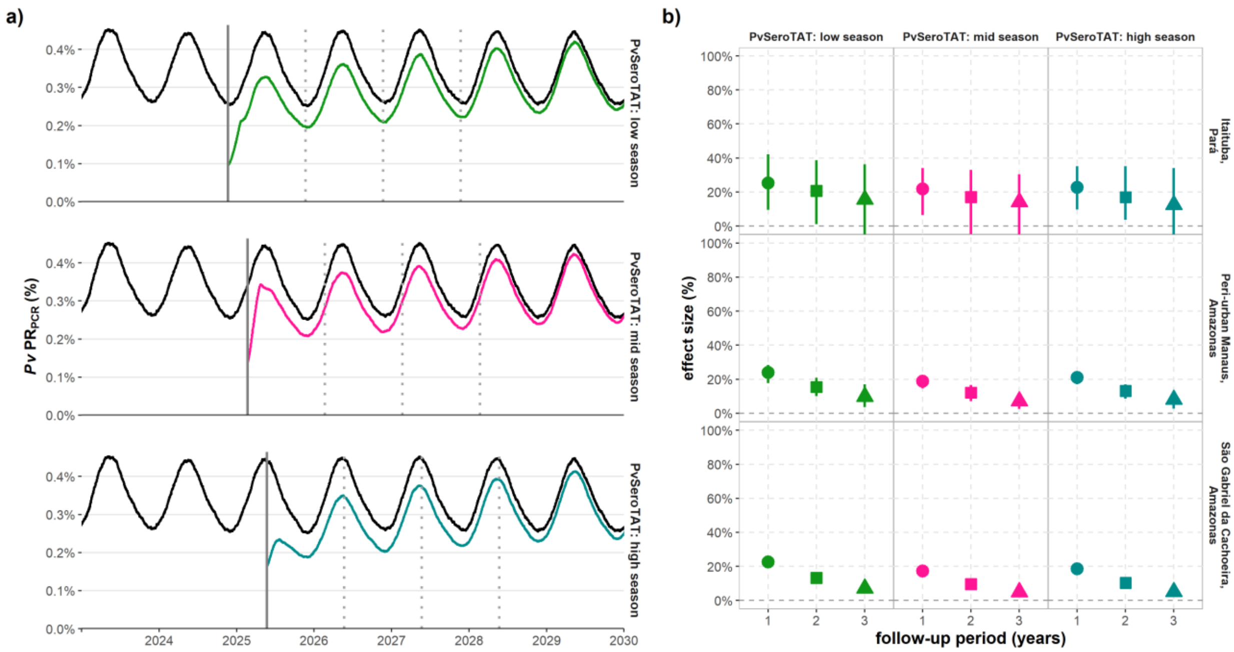

Figure S2. Impact of varying timing of a single round of *Pv*SeroTAT during the transmission season. **(A)** Model predicted *Pv*PR$_{PCR}$ over time with interventions deployed at the end of 2024 during the low transmission period, between the low and high transmission period (mid season), and during the high peak transmission period. Evaluation of the effect size compares the percent change in point *Pv*PR$_{PCR}$ before the intervention and the point *Pv*PR$_{PCR}$ one, two, and three years post-intervention (indicated by dotted vertical lines). *Pv*PR$_{PCR}$ over time is shown for S_2 PvSeroTAT (green), S_{5a} PvSeroTAT (pink), and S_{5b} PvSeroTAT (teal). **(B)** Mean effect size (%) and 95% UI of change in point *Pv*PR$_{PCR}$ for 100 stochastic simulations per scenario evaluated at one, two, and three-years follow-up.

Table S4. Clinical cases averted per 100,000 population and 95% uncertainty interval of all scenarios across archetype settings over 12 months follow-up. Values are means of 100 scenario stochastic simulations rounded to the nearest 100.

	Intervention	*Rounds*	*Interval between rounds (months)*	*Itaituba, Pará*	*Peri-urban Manaus, Amazonas*	*São Gabriel da Cachoeira, Amazonas*
S_0	Baseline CM			-100<br>[-600-600]	-200<br>[-1200-800]	-200<br>[-1400-1000]
S_1	Strengthened CM			300<br>[-300-1000]	1700<br>[500-3100]	3900<br>[1600-5900]
S_2	*Pv*SeroTAT	1		900<br>[0-1500]	3500<br>[900-6300]	7000<br>[900-13900]
S_{2a}	*Pv*SeroTAT	1		600<br>[-100-1300]	2600<br>[600-4600]	5100<br>[200-10700]
S_{2b}	*Pv*SeroTAT	1		1000<br>[100-1900]	4200<br>[1300-7000]	8200<br>[1700-15500]
S_{3a}	*Pv*SeroTAT	2	6	1300<br>[400-2200]	5500<br>[2000-8800]	10300<br>[2200-19300]
S_{3b}	*Pv*SeroTAT	2	12	1400<br>[100-2400]	5300<br>[1500-8700]	9300<br>[1300-18100]
S_{3c}	*Pv*SeroTAT	3	6	1800<br>[900-2700]	7000<br>[3100-11100]	13000<br>[3000-23800]
S_{3d}	*Pv*SeroTAT	3	12	1700<br>[400-2700]	5900<br>[1800-9800]	9700<br>[600-19800]
S_{3e}	*Pv*SeroTAT	4	6	2100<br>[1100-3000]	7500<br>[3300-11700]	13300<br>[2500-24900]
S_{3f}	*Pv*SeroTAT	4	12	1900<br>[600-3000]	6100<br>[1900-10400]	9100<br>[-400-19900]
S_4	MDA	1		1200<br>[100-1900]	4800<br>[1700-8100]	9300<br>[1900-17400]
S_{4a}	MDA	2	6	1800<br>[500-2600]	7100<br>[3100-11000]	13300<br>[3500-23800]
S_{4b}	MDA	2	12	1800<br>[700-2900]	6900<br>[2800-11100]	12300<br>[2700-22800]
S_{4c}	MDA	3	6	2300<br>[1200-3200]	8900<br>[4200-13400]	16600<br>[4800-28900]
S_{4d}	MDA	3	12	2100<br>[800-3300]	7600<br>[3000-12300]	13000<br>[1800-24800]
S_{4e}	MDA	4	6	2600<br>[1200-3400]	9400<br>[4600-14100]	16900<br>[3800-30200]
S_{4f}	MDA	4	12	2400<br>[1100-3500]	7800<br>[2800-12800]	12300<br>[-100-25300]
S_{5a}	*Pv*SeroTAT mid-season	1		700<br>[-100-1400]	3000<br>[800-5100]	5900<br>[800-11400]
S_{5b}	*Pv*SeroTAT high season	1		800<br>[100-1500]	3500<br>[900-5800]	6800<br>[1000-12900]
S_{6a}	$S_1 + S_{3a}$	5 years + 2 rounds	6	1700<br>[500-2600]	6800<br>[3700-9900]	13000<br>[5800-21100]
S_{6b}	$S_1 + S^{3e}$	5 years + 4 rounds	6	2200<br>[900-3200]	8500<br>[4400-12500]	15800<br>[5900-26300]

Table S5. Mean *Pv*PR$_{PCR}$ effect size (%) and 95% uncertainty interval of 100 stochastic simulations, and total chloroquine courses, primaquine courses, primaquine overtreatment, and G6PD tests in 100,000 at 12-month follow-up (except for S_1 over five years).

	Scenario	*Effect size*	*Chloroquine courses*	*Primaquine courses*	*Primaquine overtreatment*	*G6PD tests*
Itaituba, Pará	S_1<br>1 year	5.4%<br>[-10.7%-18.3%]	0.02<br>[0.02-0.02]	0.02<br>[0.02-0.02]	0	0.02<br>[0.02-0.02]
	S_1<br>5 years	10.8%<br>[-4.8%-28.3%]	0.11<br>[0.1-0.11]	0.09<br>[0.08-0.1]	0	0.1<br>[0.1-0.11]
	S_2	25.2%<br>[9.6%-42.2%]	0.19<br>[0.19-0.19]	0.15<br>[0.15-0.15]	0.14<br>[0.14-0.14]	0.17<br>[0.17-0.17]
	S_{3a}	38.1%<br>[24.4%-49.4%]	0.37<br>[0.37-0.37]	0.31<br>[0.31-0.31]	0.27<br>[0.27-0.27]	0.34<br>[0.34-0.34]
	S_{3c}	53.7%<br>[40.4%-65.8%]	0.56<br>[0.55-0.56]	0.46<br>[0.46-0.46]	0.41<br>[0.41-0.41]	0.5<br>[0.5-0.51]
	S_{3e}	57.5%<br>[43.6%-73.1%]	0.74<br>[0.74-0.74]	0.61<br>[0.61-0.61]	0.54<br>[0.54-0.54]	0.67<br>[0.67-0.67]
	S_4	34.4%<br>[24.9%-44%]	0.81<br>[0.81-0.81]	0.7<br>[0.7-0.7]	0.68<br>[0.68-0.68]	0.77<br>[0.77-0.77]
	S_{4a}	49.3%<br>[37.1%-62.8%]	1.62<br>[1.62-1.62]	1.4<br>[1.4-1.4]	1.36<br>[1.36-1.36]	1.55<br>[1.55-1.55]
	S_{4c}	64.8%<br>[54.9%-77.2%]	2.43<br>[2.43-2.43]	2.1<br>[2.1-2.1]	2.04<br>[2.04-2.04]	2.32<br>[2.32-2.32]
	S_{4e}	70.2%<br>[55.5%-80.6%]	3.25<br>[3.24-3.25]	2.8<br>[2.8-2.8]	2.72<br>[2.72-2.73]	3.09<br>[3.09-3.09]
peri-urban Manaus, Amazonas	S_1<br>1 year	5.3%<br>[0.1%-11.2%]	0.1<br>[0.1-0.11]	0.09<br>[0.09-0.09]	0	0.1<br>[0.1-0.1]
	S_1<br>5 years	11%<br>[4.4%-17.8%]	0.5<br>[0.48-0.52]	0.43<br>[0.42-0.45]	0	0.48<br>[0.46-0.5]
	S_2	24%<br>[17.8%-28.4%]	0.23<br>[0.23-0.23]	0.2<br>[0.2-0.2]	0.13<br>[0.13-0.13]	0.21<br>[0.21-0.21]
	S_{3a}	33.9%<br>[29.7%-38.4%]	0.47<br>[0.47-0.47]	0.4<br>[0.4-0.4]	0.25<br>[0.25-0.25]	0.43<br>[0.43-0.43]
	S_{3c}	46.9%<br>[43.3%-51.9%]	0.69<br>[0.69-0.69]	0.59<br>[0.59-0.59]	0.38<br>[0.38-0.38]	0.63<br>[0.63-0.64]
	S_{3e}	47.6%<br>[42.7%-51.2%]	0.9<br>[0.9-0.9]	0.77<br>[0.77-0.77]	0.51<br>[0.51-0.51]	0.83<br>[0.83-0.83]
	S_4	31.2%<br>[27.1%-36.4%]	0.81<br>[0.81-0.81]	0.72<br>[0.72-0.72]	0.63<br>[0.63-0.63]	0.77<br>[0.77-0.77]
	S_{4a}	42.7%<br>[38.4%-46.6%]	1.62<br>[1.62-1.62]	1.44<br>[1.44-1.44]	1.26<br>[1.26-1.26]	1.55<br>[1.55-1.55]
	S_{4c}	58.2%<br>[53.8%-61.9%]	2.43<br>[2.43-2.43]	2.16<br>[2.16-2.16]	1.91<br>[1.91-1.91]	2.32<br>[2.32-2.32]
	S_{4e}	57.9%<br>[53.5%-62.6%]	3.25<br>[3.24-3.25]	2.88<br>[2.87-2.88]	2.57<br>[2.57-2.57]	3.09<br>[3.09-3.09]
São Gabriel da Cachoeira, Amazonas	S_1<br>1 year	4.8%<br>[2.2%-7.4%]	0.24<br>[0.24-0.25]	0.22<br>[0.22-0.23]	0	0.23<br>[0.23-0.24]
	S_1<br>5 years	9.1%<br>[6.3%-11.8%]	1.18<br>[1.15-1.2]	1.08<br>[1.05-1.11]	0	1.13<br>[1.11-1.16]
	S_2	22.5%<br>[20.2%-24.8%]	0.35<br>[0.35-0.35]	0.31<br>[0.31-0.32]	0.09<br>[0.09-0.09]	0.33<br>[0.33-0.33]
	S_{3a}	30%<br>[27.6%-32.9%]	0.7<br>[0.7-0.7]	0.63<br>[0.63-0.63]	0.19<br>[0.19-0.19]	0.66<br>[0.66-0.66]
	S_{3c}	44.5%<br>[42.3%-46.2%]	1.02<br>[1.02-1.02]	0.92<br>[0.92-0.92]	0.29<br>[0.29-0.29]	0.96<br>[0.95-0.96]
	S_{3e}	41.4%<br>[39.2%-43.7%]	1.33<br>[1.33-1.33]	1.19<br>[1.19-1.19]	0.4<br>[0.4-0.4]	1.24<br>[1.24-1.24]
	S_4	29.1%<br>[26.8%-31.5%]	0.81<br>[0.81-0.81]	0.74<br>[0.74-0.74]	0.47<br>[0.47-0.47]	0.77<br>[0.77-0.77]
	S_{4a}	37.9%<br>[35.7%-40%]	1.62<br>[1.62-1.62]	1.48<br>[1.48-1.48]	0.94<br>[0.94-0.94]	1.54<br>[1.54-1.55]

S_{4c}	54.5% [52.6%-56.4%]	2.43 [2.43-2.43]	2.22 [2.22-2.22]	1.48 [1.47-1.48]	2.32 [2.32-2.32]
S_{4e}	51.5% [48%-53.8%]	3.24 [3.24-3.25]	2.96 [2.96-2.97]	2.04 [2.04-2.04]	3.09 [3.09-3.09]

Since testing and treatment administration through CM relies on only symptomatic clinical cases seeking care, only a small proportion of infections will receive radical cure (Table S5). For MDA campaigns, all targeted individuals over six months old and not pregnant will receive a chloroquine course, be tested for G6PD activity and if G6PD normal receive a primaquine course with or without a detectable *P. vivax* infection. Therefore, in all modelled settings, we observe total courses per MDA round equivalent to 80% of the population minus those ineligible for treatment. For *Pv*SeroTAT campaigns on the other hand, individuals are first screened for previous *P. vivax* infection with the serological diagnostic before subsequent treatment and G6PD testing among only seropositive individuals.

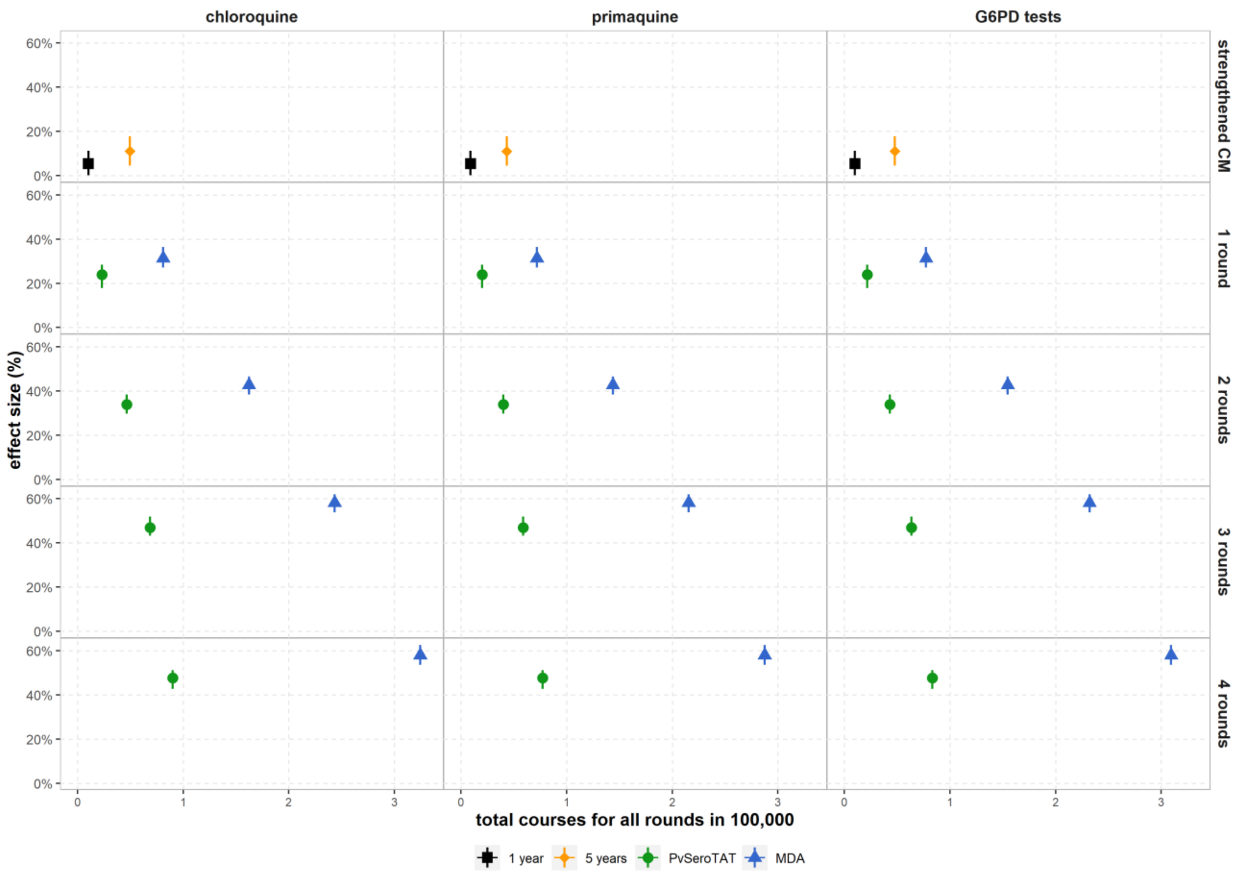

Figure S3. Total treatment courses and G6PD tests in 100,000 for scenarios by mean *Pv*PR$_{PCR}$ effect size at 12 months in peri-urban Manaus, Amazonas. For strengthened CM, we show the cumulative number of courses and tests one or five years after deployment and the effect size at year one or year five respectively. We show results for multiple rounds of *Pv*SeroTAT and MDA interventions six months apart for two, three, or four rounds and effect size at one-year follow-up. No significant difference in the total number of courses or tests is observed if rounds are six months or 12 months apart. Each scenario was modelled with a population size of 100,000 individuals.

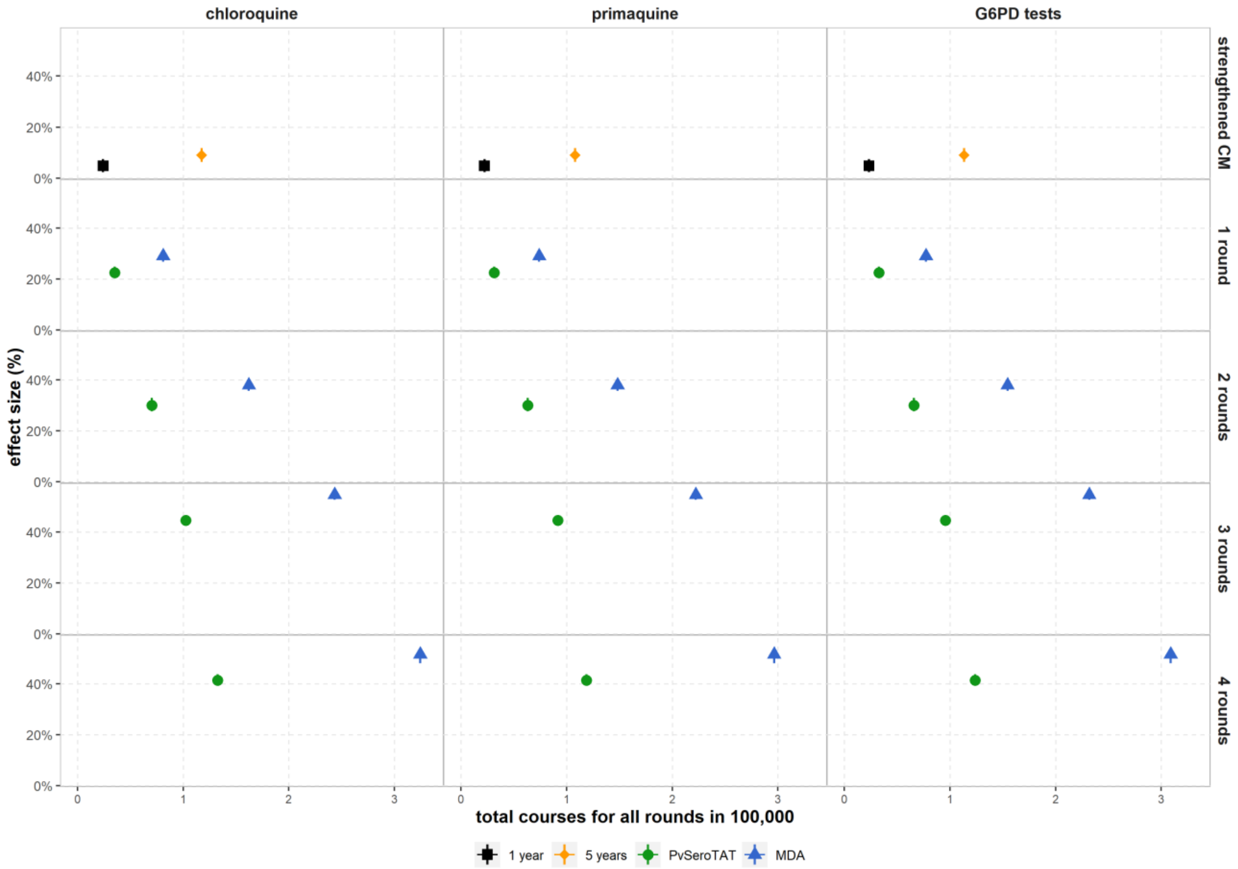

Figure S4. Total treatment courses and G6PD tests in 100,000 for scenarios by mean *Pv*PR$_{PCR}$ effect size at 12 months in São Gabriel da Cachoeira, Amazonas. For strengthened CM, we show the cumulative number of courses and tests one or five years after deployment and the effect size at year one or year five respectively. We show results for multiple rounds of *Pv*SeroTAT and MDA interventions six months apart for two, three, or four rounds and effect size at one-year follow-up. No significant difference in the total number of courses or tests is observed if rounds are six months or 12 months apart. Each scenario was modelled with a population size of 100,000 individuals.

Table S6. Mean and 95% uncertainty intervals (UI) of point *Pv*PR_{PCR} reduction in simulated municipalities (n=126) by baseline simulation incidence per 1,000 population for S_{6a} and S_{6b}.

Baseline incidence per 1000	***Municipalities (%)***	***S_{6a}* Mean and 95% UI**			***S_{6b}* Mean and 95% UI**		
		12 months	24 months	36 months	12 months	24 months	36 months
0 to 5	14 (11.1%)	72.4% [44.8%-86%]	72.6% [46.1%-88.5%]	75.3% [46.2%-91.1%]	83.6% [70.6%-94.9%]	85.4% [71.3%-95.2%]	86.2% [71.3%-95.9%]
5 to 10	16 (12.7)	58.7% [45.6%-72.5%]	59.4% [42.7%-76.5%]	60.3% [42.5%-78.1%]	74.1% [61.3%-86.3%]	74.2% [59.3%-86.9%]	73.4% [56.5%-87.2%]
10 to 20	30 (23.8%)	53.6% [44%-64.1%]	51.7% [39.3%-64.7%]	50.2% [33.5%-66.4%]	69.5% [59.5%-79.4%]	66.9% [53.1%-80.2%]	63.9% [46.8%-80.8%]
20 to 50	24 (19%)	48.2% [28.9%-59.8%]	44.5% [22.8%-58.9%]	41.2% [17.1%-58.9%]	65.2% [46.8%-75.4%]	60.3% [39%-74.4%]	55.1% [31.1%-73.3%]
50 to 100	19 (15.1%)	42.4% [35.8%-50.8%]	34.9% [26.4%-46.4%]	28.6% [19.6%-41.5%]	57.5% [48.3%-67.2%]	47.2% [35.2%-61.4%]	37.6% [24.6%-54.8%]
100 to 200	15 (11.9%)	40.1% [35.5%-45.1%]	30.9% [25.3%-38%]	23.8% [18.1%-31.8%]	54% [47.5%-60.2%]	41.5% [33%-50.9%]	30.8% [22.6%-41.3%]
200 to 500	6 (4.8%)	23.5% [11.8%-33.2%]	13.7% [4.9%-21.5%]	8.4% [2.5%-14.1%]	30.3% [13.6%-44.4%]	17.1% [5.3%-28%]	9.9% [2.5%-17.3%]

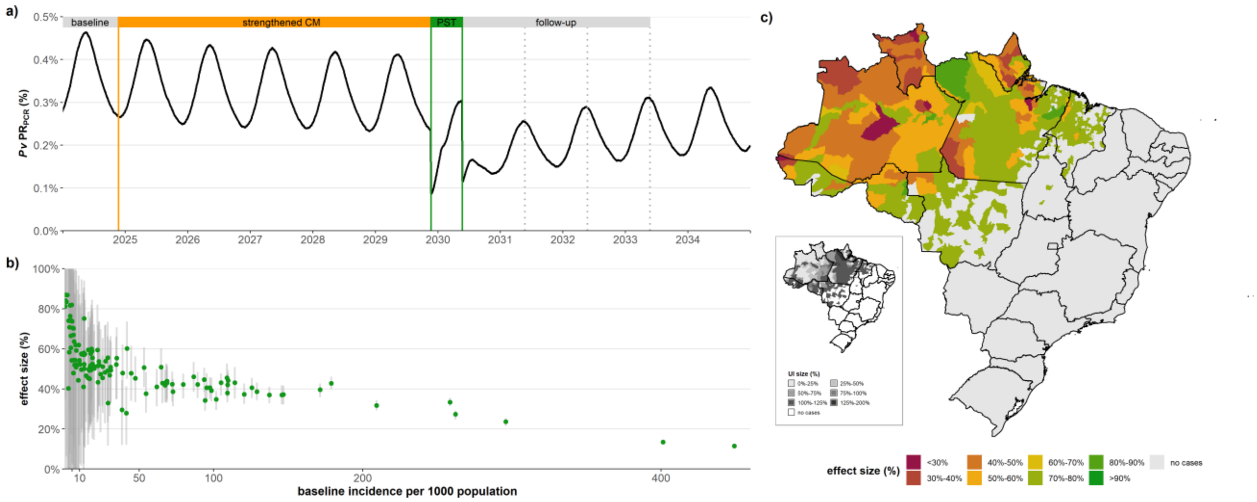

Figure S5. Impact of combined strengthened case management followed by two rounds of *Pv*SeroTAT mass campaigns deployed six months apart. **(A)** *Pv*PR_{PCR} over time in Itaituba. Strengthened CM starts indefinitely at the end of 2024 (yellow vertical line). After five years, two rounds of *Pv*SeroTAT campaigns deployed six months apart (green vertical lines) are deployed. The effect size or relative change in point *Pv*PR_{PCR} is measured 12, 24, and 36 months after the last round (dotted vertical lines). **(B)** The mean point *Pv*PR_{PCR} effect size at 12 months (green point) and the 95% UI (grey vertical bars) are shown for all simulated (# municipalities = 126) by the baseline simulation incidence per 1,000 population. **(C)** Each setting's mean point *Pv*PR_{PCR} effect size at 12 months is mapped by colour in the main plot and the size of the 95% UI is shown in the inset plot. For the simulated settings (n=126), estimates of mean effect size are from model simulations. For the non-simulated settings, we assumed a mean effect from the simulated settings with a similar 2018 baseline incidence: 295 settings had an incidence of less than five per 1,000 population which corresponded to a mean effect size of 72.4% [95% UI: 44.8%-86%] at 12 months in the simulated municipalities; two settings had an incidence between five and 10 cases per 1,000 population which corresponded to a mean point prevalence effect size of 58.7% [95% UI: 45.6%-72.5%] at 12 months in the simulated settings; one setting had an incidence between 10 and 20 cases per 1,000 population which corresponded to a mean point prevalence effect size of 53.6% [95% UI: 44%-64.1%] at 12 months in the simulated settings (Table S6).

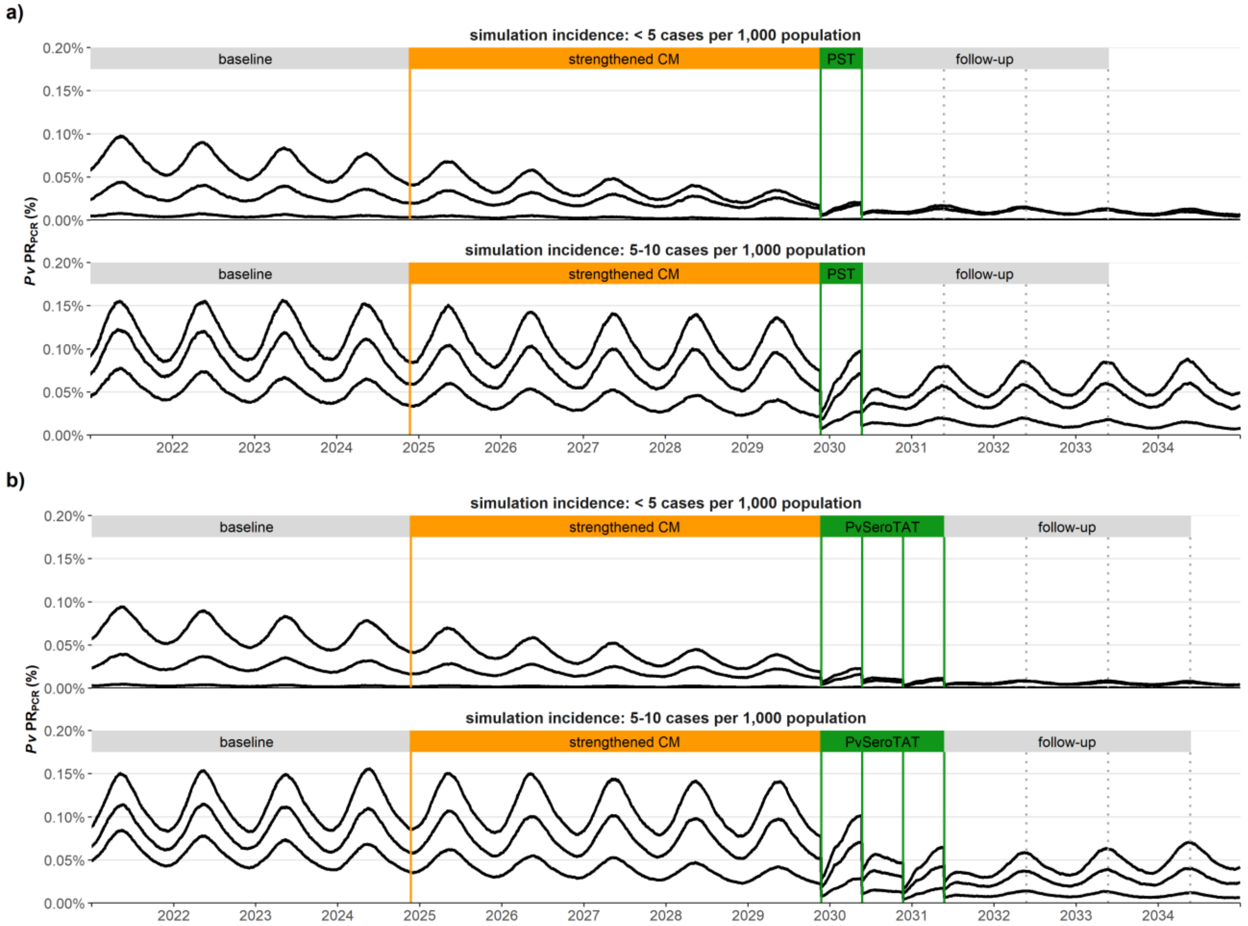

Figure S6. *Pv*PR$_{PCR}$ over time in six example simulated municipalities with low incidence. Settings are separated by baseline simulated incidence: three example municipalities with < 5 cases per 1,000 population and three example municipalities with 5 to 10 cases per 1,000 population. The effect size or relative change in point *Pv*PR$_{PCR}$ is measured 12, 24, and 36 months after the last round (dotted vertical lines). For very low transmission settings with a baseline < 5 cases per 1,000 population, we observe less stable baseline transmission and therefore, less reliable effect size estimates (Table S6). In these settings, stochastic noise and fadeout result in more unstable transmission dynamics and greater variation between simulations. In addition, as in all modelled settings, we assume homogenous mixing in the population and no importations to sustain transmission; therefore, local transmission dynamics in communities are likely to differ compared to the simplified model. Simulated municipalities with incidence > 5 cases per 1,000 had less fadeout and were more stable. **(A)** Follow-up of impact on *Pv*PR$_{PCR}$ with S$_{6a}$. **(B)** Follow-up of impact on *Pv*PR$_{PCR}$ with S$_{6b}$.
